# Full genome sequence for the African swine fever virus outbreak in the Dominican Republic in 1980

**DOI:** 10.1038/s41598-022-25987-5

**Published:** 2023-01-19

**Authors:** Edward Spinard, Vivian O’Donnell, Elizabeth Vuono, Ayushi Rai, Charronne Davis, Elizabeth Ramirez-Medina, Nallely Espinoza, Alyssa Valladares, Manuel V. Borca, Douglas P. Gladue

**Affiliations:** 1 Plum Island Animal Disease Center, Agricultural Research Service, USDA, Greenport, NY 11944 USA; 2 Plum Island Animal Disease Center, Animal and Plant Health Inspection Service, USDA, Greenport, NY 11944 USA; 3 Department of Pathobiological Sciences, School of Veterinary Medicine, University of Wisconsin-Madison, Madison, WI USA; 4 Oak Ridge Institute for Science and Education (ORISE), Oak Ridge, TN 37830 USA; 5 ATCC Federal Solutions, ATCC, 10801 University Blvd, Manassas, VA 20110 USA

**Keywords:** Microbiology, Molecular biology

## Abstract

African swine fever is a lethal disease of domestic pigs, geographically expanding as a pandemic, that is affecting countries across Eurasia and severely damaging their swine production industry. After more than 40 years of being absent in the Western hemisphere, in 2020 ASF reappeared in the Dominican Republic and Haiti. The recent outbreak strain in the Dominican Republic has been identified as a genotype II ASFV a derivative of the ASF strain circulating in Asia and Europe. However, to date no full-length genome sequence from either the 1978–1980 Here we report the complete genome sequence of an African swine fever virus (ASFV) (DR-1980) that was previously isolated from blood collected in 1980 from the Dominican Republic at the end of the last outbreak, before culling of all swine on the island of Hispaniola and stored in the Plum Island Animal Disease Center ASFV repository. A contig representing the full-length genome (183,687 base pairs) was de novo assembled into a single contig using both Nanopore and Illumina sequences. DR-1980 was determined to belong to genotype I and, as determined by full genome comparison, a close relative to the sequenced Sardinia viruses that were causing outbreaks at this time.

## Introduction

Currently ASFV, the etiological agent for African swine fever (ASF) is causing a pandemic in swine, that has been mostly restrained to the Eastern hemisphere. However, in 2021 for the first time the pandemic strain of ASF was detected in the Dominican Republic ASF and has caused continued outbreaks on the island of Hispaniola^1^. Currently, there is only one vaccine for commercial use for ASF and its use is limited in Vietnam, with several other experimental vaccines under different stages of development^2^. For the rest of the world, control of ASF is restricted to culling of infected farms and animals.

African swine fever virus (ASFV) is large dsDNA virus, that encodes for a large number of genes The exact number of genes present in the genome as well as the genome length (ranging from 170 to 190 kb) vary among different virus isolates. The ASFV genome encodes for over 150 different proteins, with variation between isolates, particularly in the multi-gene family genes, and genes that have been used to genotype ASFV: P54, P72, and the central variable region (CVR) of pB602L^3^. Until recently full-length sequencing the large genome of ASFV was costly and difficult, which restricted this type of information to very few historical genomes of ASFV that have been fully sequenced. The lack of historical ASFV isolates with full sequenced genomes has been identified as a major GAP in ASFV research^4^ as this knowledge is of great importance in understanding historical outbreaks of ASFV, and the evolution of the ASFV genome over time, particularly in variable regions of the virus genome.

The 2021 outbreak in the Dominican Republic was caused by an isolate that has been determined to be a derivative of the pandemic strain currently circulating in Asia and Europe, and is a genotype II ASFV^5^. Here we report, for the first time, the full-length sequence of the African swine fever strain that caused the outbreak in 1978–180 in the Dominican Republic, and determined that it was a close relative to the sequenced Sardinia viruses that were causing outbreaks at that time^6,7^.

## Results

### ASFV full genome alignment

**Figure 1:**
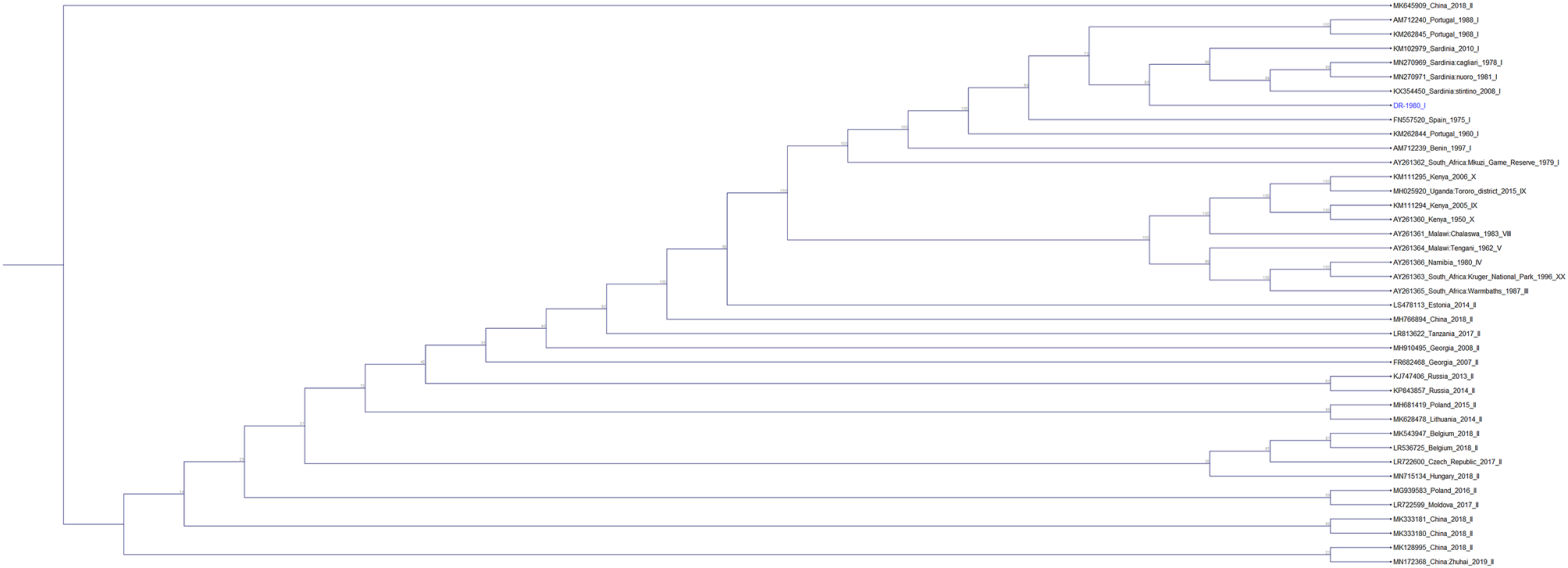
The full-length trees. 40 curated ASFV sequences were aligned to DR-1980 and a cladogram was created using the neighbor joining method and Jukes-Cantor mode with phylogeny inferred using 1000 bootstraps. Bootstrap values are shown at each branch. Genomes are labeled by GenBank accession number region of isolation date of isolation_p72 genotype. DR-1980 is highlighted in blue font*.*

Forty curated ASFV sequences were aligned to DR-1980 using the default parameters of Genome Alignment tool in CLC Workbench. A Cladogram was created with representative sequences using the neighbor joining method and Jukes-Cantor mode. Phylogeny was inferred using 1000 bootstrap replications showing that the DR-1980 isolate was most closely related to the ASFV genomes that were sequenced form outbreaks occurring in Sardinia at that time (Fig. 1).

### Genotyping of DR-1980

Genotyping of the DR-1980 isolate was determined following the three commonly used methods^3^ (Table 1). ASFV isolate 56/Ca/1978 (Sardinia: Cagliari-1978), which was circulating during the same time frame, was 99% identical and the closest match based on whole genome sequence identity to DR-1980. Of note, very few full-length ASFV genomes have been sequenced from this time, therefore the possible origin of DR-1980 should not be overstated and could have been from any outbreak region especially considering the two viruses are not genetically identical.

**Table 1:** Genotyping of DR-1980.

Genotyping of DR-1980	
Gene	Genotype
p72/VP73 (B646L)	I
CVR (9RL/B602L)	Tet-25a
p54 (E183L)	Ia

### Analysis of individual DR-1980 proteins

The 165 annotations of DR-1980 were translated in CLC Genomics Workbench and extracted. ASFV [Taxonomy ID: 10497] nucleotide sequences were downloaded from the International Nucleotide Sequence Database Collaboration (INSDC) using the National Center for Biotechnology Information (NCBI) website (http://www.ncbi.nlm.nih.gov) in GenBank and fasta format (accessed March 14th, 2022). All protein coding sequences (CDS) were extracted and combined with ASFV protein coding sequences downloaded from the Viral Bioinformatics Research Centre (VBRC) website (https://4virology.net) (accessed April 13th, 2022) to create a local ASFV database that was compared to the DR-1980 translations using the default parameters of blastp.

The following genes encoded for unique nucleotide sequences, specific for DR-1980: KP93L (truncated form of the protein, 85% identical to closest sequenced isolate), DP93R (unique amino acid P45A), L60L (unique amino acid D3N), I215L (D203del), MGF 360-12L (unique amino acid combination of R101Q or M151L or N191S & S176N), G1211R (K505R), and CP2475L (unique amino acid P204A). Amino acid sequences for each of the 7 unique DR-1980 genes were aligned to other sequenced isolates (supplemental Fig. 1) with the unique amino acids for the DR-1980 isolate highlighted.

## Discussion

Currently the island of Hispaniola is facing a continued outbreak from a descendent of the Georgia 2007 strain that is causing the continued outbreaks in Asia and Europe. Here we report, for the first time, a full-length sequence from the outbreak in 1978–1980 in the Dominican Republic, addressing the historical GAP in available full-length ASFV genomes^4^. The conclusion of this outbreak was to cull all the swine on the island of Hispaniola and repopulate. At the time of this outbreak there was no vaccine available for ASFV, and control was limited only to culling of infected animals. Recently, the first ASF commercial vaccine was approved that is based on the backbone of the current genotype II pandemic strain containing a single deletion in the I177L gene^8^. This vaccine could be considered to help in the control of the current outbreak situation in the island of Hispaniola. However, the possibility of controlling ASF outbreaks with the aid of a vaccination program, was not an available option in 1980. The decision in 1980 was made to cull all swine from the island of Hispaniola to stop continued outbreaks of ASF. The full-length sequence of the 1980 strain is valuable information that could aid in making different decisions for the current outbreak in the Dominican Republic.

## Methods

### Viral DNA isolation and next generation sequencing

ASFV isolate DR-180 was passed once in primary swine macrophage cultures produced from blood, as previously described^9^. Viral DNA was sequenced, as previously described, using an Illumina Nextseq500^10^ and oxford nanopore MinIon^11^ sequencing platforms. In brief, virus DNA was extracted from infected macrophage cultures using the MagMax pathogen RNA/DNA kit (Applied Biosystems). For nanopore sequencing, subtractive hybridization of methylated genomic DNA was performed, the DNA was barcoded using the rapid PCR barcoding kit, and loaded per manufacture instructions onto the GridIon. For illumina sequencing, the Nextera XT v2 kit was used (Illumnia, San Diego, CA, USA) following the manufacturer’s protocol. Sequence analysis was performed using CLC Genomics Workbench software (CLCBio, Waltham, MA, USA). Coverage Plots of reads mapped back to the consensus sequence (supplemental Fig. 2).

### Genome assembly

All steps were performed using CLC Genomics Workbench (version 21). Illumina reads were trimmed for quality (limit = 0.05), ambiguous base pairs (max = 2), adapters, minimum size (min = 50) and from the 5′ (20 nucleotides) and 3′ terminal end (5 nucleotides). To remove reads resulting from host sequence, Minion and Illumina reads were mapped to ASFV strain Nu1979 (Accession: MW723481.1) and collected, resulting in 377 Minion reads, 17 million paired-end Illumina reads, and 7,885 orphaned Illumina reads. All reads were then entered into the “De Novo Assemble Long Reads and Polish with Short Reads” pipeline using the default parameters resulting in four contigs. De novo assembly was then performed using only the Illumina reads, with the four polished contigs serving as guidance only reads. 5 contigs were created. Both assemblies were combined and one 183, 687 bp contig was constructed based on homology overlap between the contigs. Illumina reads were mapped backed to the genome using default parameters and resulted in an average depth of coverage of 10,529 reads.

### Annotation of the genome

All 165 translated protein sequences were extracted from the ASFV strain 56/Ca/1978, (Accession: MN270969.1) GenBank file and compared against the DR-1980 consensus sequence using the default parameters of tblastn. Strand, start and end nucleotide positions for each gene were extracted from the output file and, when required, manually extended to include the correct start and stop codon. Annotations were entered on to the DR-1980 genome in CLC Genomics Workbench, from ASFV reference genomes resulting in 165 annotations.

## Supplementary Information

Supplementary Figure 1.

Supplementary Figure 2.

## Data Availability

This genome sequence has been deposited in GenBank under the accession no ON185726. Raw data for this project can be found in the GenBank SRA under accession no. SRX16252875 to SRX16252901 in BioProject accession no. PRJNA859002.

## References

[1] Costard S, Wieland B, de Glanville W, Jori F, Rowlands R, Vosloo W, Roger F, Pfeiffer DU, Dixon LK (2009). African swine fever: How can global spread be prevented?. Philos. Trans. R. Soc. Lond. B Biol. Sci..

[2] Gladue DP, Borca MV (2022). Recombinant ASF live attenuated virus strains as experimental vaccine candidates. Viruses.

[3] Qu H, Ge S, Zhang Y, Wu X, Wang Z (2022). A systematic review of genotypes and serogroups of African swine fever virus. Virus Genes.

[4] Global African Swine REsearch Alliance. *Global African Swine Research Alliance 2018 Gap Analysis*. https://www.ars.usda.gov/gara/reports/GARAGapAnalysisReport2018.pdf. Accessed 14 Nov 2022 (2018).

[5] Gonzales W, Moreno C, Duran U, Henao N, Bencosme M, Lora P, Reyes R, Nunez R, De Gracia A, Perez AM (2021). African swine fever in the Dominican Republic. Transbound. Emerg. Dis..

[6] Bastos AD, Penrith ML, Cruciere C, Edrich JL, Hutchings G, Roger F, Couacy-Hymann E (2003). G RT: Genotyping field strains of African swine fever virus by partial p72 gene characterisation. Adv. Virol..

[7] Torresi C, Fiori M, Bertolotti L, Floris M, Colitti B, Giammarioli M, DeiGiudici S, Oggiano A, Malmberg M, DeMia GM (2020). The evolution of African swine fever virus in Sardinia (1978–2014) as revealed by whole-genome sequencing and comparative analysis. Transbound. Emerg. Dis..

[8] Borca MV, Ramirez-Medina E, Silva E, Vuono E, Rai A, Pruitt S, Holinka LG, Velazquez-Salinas L, Zhu J, Gladue DP (2020). Development of a highly effective African swine fever virus vaccine by deletion of the I177L gene results in sterile immunity against the current epidemic Eurasia strain. J. Virol..

[9] Borca MV, Berggren KA, Ramirez-Medina E, Vuono EA, Gladue DP (2018). CRISPR/Cas gene editing of a large DNA virus: African swine fever virus. Bio-Protoc..

[10] Borca MV, Holinka LG, Berggren KA, Gladue DP (2018). CRISPR-Cas9, a tool to efficiently increase the development of recombinant African swine fever viruses. Sci. Rep..

[11] O'Donnell VK, Grau FR, Mayr GA, Sturgill Samayoa TL, Dodd KA, Barrette RW (2019). Rapid sequence-based characterization of African swine fever virus by use of the Oxford nanopore MinION sequence sensing device and a companion analysis software tool. J. Clin. Microbiol..

