# Supplementary material for: Full genome sequence for the African swine fever virus outbreak in the Dominican Republic in 1980
Source: Sci Rep. 2023 Jan 19;13:1024. doi: 10.1038/s41598-022-25987-5 (PMC9852453; doi:10.1038/s41598-022-25987-5)
Supplement: Supplementary file 1 — Supplementary Figure 1. [file 41598_2022_25987_MOESM1_ESM.pdf]

CP2475L Alignment

```
                                        20         40         60         80
CP2475L_DR-1980     MGNRGSSTSS RPPLSSEANL YAKLQDHIQR QTRPFSGGGY FNGGGDKNPV QHIKDYHIDS VSSKAKLRVI EGIIRAIAKI  80
LO2018_minor        .......... .......... .......... .......... .......... .......... .......... ..........  80
31479_2015          .......... .......... .......... .......... .......... .......... .......... ..........  80
OR1984              .......... .......... .......... .......... .......... .......... .......... ..........  80
Benin_97/1          .......... .......... .......... .......... .......... .......... .......... ..........  80
15998_2015          .......... .......... .......... .......... .......... .......... I......... ..........  80
Pig/SD/DY-I/2021    .......... .......... .......... .......... .......... .......... .......... ..........  80
Warmbaths           .......... ...P.....I .......... .......... .......... .......... ........I. ..........  80
Mkuzi_1979          .......... ...P.....I .......... .......... .......... .......... ........I. ..........  80
Tengani_62          .......I.. .........I .......... .......... .......... .......... ........I. ..........  80
ASFV-G              .......... ..LP.....I .......... .......... .......... .......... ........I. ..........  80
Ulyanovsk_19/WB-5699 ......... ..LP.....I .......... .......... .......... .......... ........I. ..........  80
HuB20               .......... ..LP.....I .......... .......... .......... .......... ........I. ..........  80
Pretorisuskop/96/4  .......... ...P.....I .......... .......... .......... .......... ........I. ..........  80
Warthog             .......... ...P.....I .......... .......... .......... .......... ........I. ..........  80
K49                 .......... .......... .......... .......... .......... .......... .......... ..........  80
OmLF2               .......... ..LP.....I .......... .......... .......... .......... ........I. ..........  80
Malawi_Lil-20/1     .......... .........I .......... .......... .......... .......... ........I. ....K..S..  80
Ken05/Tk1           .......... .......... .......... .....A.... .......... .......... ........I. ....K..S..  80
Kenya_1950          .......... .......... .......... .......... .......... .......... ........I. ....K..S..  80
Ken.rie1            .........T .......... .......... .......... .......... .......... ........I. ....K..S..  80
Uvira_B53           .......... .......... .......... .......... .......... .......... ........I. ....K..S..  80
Ken06.Bus           .......... .......... .......... ...S...... .......... .......... ........I. ....K..S..  80
N10                 .......... .......... .......... ...S...... .......... .......... ........I. ....K..S..  80
Conservation

                                       100        120        140        160
CP2475L_DR-1980     GFKVDTKQPI EDILKDIKKQ LPDPRAGSTF VKNAEKQETV CKMIADAINQ EFIDLGQDKL IDTTDGAASI CRQIVLYINS  160
LO2018_minor        .......... .......... .......... .......... .......... .......... .......... ..........  160
31479_2015          .......... .......... .......... .......... .......... .......... .......... ..........  160
OR1984              .......... .......... .......... .......... .......... .......... .......... ..........  160
Benin_97/1          .......... .......... .......... .......... .......... .......... .......... ..........  160
15998_2015          .......... .......... .......... .......... .......... .......... .......... ..........  160
Pig/SD/DY-I/2021    .......... .......... .......... .......... .......... .......... .......... ...L......  160
Warmbaths           .......... .......... .......... .......... .......... .......... .......... ..........  160
Mkuzi_1979          .......... .......... .......... .......... .......... .......... .......... ..........  160
Tengani_62          .......... .......... .......... .......... .......... .......... ....E..... ..........  160
ASFV-G              .......... .......... .......... .......... .......... .......... ....E..... ..........  160
Ulyanovsk_19/WB-5699 ......... .......... .......... .......... .......... .......... ....E..... ..........  160
HuB20               .......... .......... .......... .......... .......... .......... ....E..... ..........  160
Pretorisuskop/96/4  .......... .......... .......... .......... .......... .......... ....E..... ..........  160
Warthog             .......... .......... .......... .......... .......... .......... ....E..... ..........  160
K49                 .......... .......... .......... .......... .......... .......... .......... ..........  160
OmLF2               .......... .......... .......... .......... .......... .......... .......... ..........  160
Malawi_Lil-20/1     ...I...... .......... .......... .......... .......... .......... ....E..... ..........  160
Ken05/Tk1           .......... .......... .......... .........I .......... .......... ....E..... ..........  160
Kenya_1950          .......... .......... .......... .........I .......... .......... ....E..... ..........  160
Ken.rie1            .......... .......... .......... .........I .......... .......... ....E..... ..........  160
Uvira_B53           .......... .......... .......... .........I .......... .......... ....E..... ..........  160
Ken06.Bus           .......... .......... .......... .........I .......... .......... ....E..... ..........  160
N10                 .......... .......... .......... .........I .......... .......... ....E..... ..........  160
Conservation

                                       180        200        220        240
CP2475L_DR-1980     LTHGLRAEYL DVHGSIENTL ENIKLLNDAI KQLHERMVTE VTKAAPNEEV INAVTMIEAV YRRLLNEQNL QINILTNFID  240
LO2018_minor        .......... .......... .......... .......... .......... .......... .......... ..........  240
31479_2015          .......... .......... .......... .......... .......... .......... .......... ..........  240
OR1984              .......... .......... .......... .......... .......... .......... .......... ..........  240
Benin_97/1          .......... .......... .......... .......... .......... .......... .......... ..........  240
15998_2015          .......... .......... .......... .......... .......... .......... .......... ..........  240
Pig/SD/DY-I/2021    .......... .......... .......... .......... .......... .......... .......... ..........  240
Warmbaths           .......... .......... .......... .......... .......... .......... .......... ..........  240
Mkuzi_1979          .......... .......... .......... .......... .......... .......... .......... ..........  240
Tengani_62          .......... .......... .......... .......... .......... .......... .......... ..........  240
ASFV-G              .......... .......... .......... .......... .......... .......... .......... ..........  240
Ulyanovsk_19/WB-5699 ......... .......... .......... .......... .......... .......... .......... ..........  240
HuB20               .......... .......... .......... .......... .......... .......... .......... ..........  240
Pretorisuskop/96/4  .......... .......... .......... .......... .......... .......... .......... ..........  240
Warthog             .......... .......... .......... .......... .......... .......... .......... ..........  240
K49                 .......... .......... .......... .......... .......... .......... .......... ..........  240
OmLF2               .......... .......... .......... .......... ....P..... .......... .......... ..........  240
Malawi_Lil-20/1     .......... .......... .......... .......... .......... .......... .......... ..S.......  240
Ken05/Tk1           .......... .......... ......S... .......... .......... .......... .......... ..........  240
Kenya_1950          .......... .......... ......S... .......... .......... .......... .......... ..........  240
Ken.rie1            .......... .......... ......S... .......... .......... .......... .......... ..........  240
Uvira_B53           .......... .......... ......S... .......... .......... .......... .......... ..........  240
Ken06.Bus           .......... .......... ......S... .......... .......... .......... .......... ..........  240
N10                 .......... .......... ......S... .......... .......... .......... .......... ..........  240
Conservation
```

CP2475L Alignment

```
                                     260                   280                   300                   320
CP2475L_DR-1980       NILTPTQKEL DKLQTDEVDI IKLLNDTNSV LGTKNFGKVL SYTLCNLGIA ASVANKINKA LQKVGLKVEQ YLQSKNWAEF  320
LO2018_minor          .......... .......... .......... .......... .......... .......... .......... ..........  320
31479_2015            .......... .......... .......... .......... .......... .......... .......... ..........  320
OR1984                .......... .......... .......... .......... .......... .......... .......... ..........  320
Benin_97/1            .......... .......... .......... .......... .......... .......... .......... ..........  320
15998_2015            .......... .......... .......... .......... .......... .......... .......... ..........  320
Pig/SD/DY-I/2021      .......... .......... .......... .......... .......... .......... .......... ..........  320
Warmbaths             .......... .......... .......... .......... .......... .......... .......... ..........  320
Mkuzi_1979            .......... .......... .......... .......... .......... .......... .......... ..........  320
Tengani_62            .......... .......... .......... .......... .......... .......... .......... ..........  320
ASFV-G                .......... .......... .......... .......... .......... .......... .......... ..........  320
Ulyanovsk_19/WB-5699  .......... .......... .......... .......... .......... .......... .......... ..........  320
HuB20                 .......... .......... .......... .......... .......... .......... .......... ..........  320
Pretorisuskop/96/4    .......... .......... .......... .......... .......... .......... .......... .......E..  320
Warthog               .......... .......... .......... .......... .......... .......... .......... ..........  320
K49                   .......... .......... .......... .......... .......... .......... .......... ..........  320
OmLF2                 .......... .......... .......... .......... .......... .......... .......... ..........  320
Malawi_Lil-20/1       .......... .......... .......... .......... .......... .......... ..R....... ..........  320
Ken05/Tk1             .......... ...K...... ..I....... .......... .......... .T........ ..R....... ..H.......  320
Kenya_1950            .......... ...K...... ..I....... .......... .......... .T........ ..R....... ..H.......  320
Ken.rie1              .......... ...K...... ..I....... .......... .......... .T........ ..R....... ..H.......  320
Uvira_B53             .......... ...K...... ..I....... .......... .......... .T........ ..R....... ..H.......  320
Ken06.Bus             .......... ...K...... ..I....... .......... .......... .T........ ..R....... ..H.......  320
N10                   .......... ...K...... ..I....... .......... .......... .T........ ..R....... ..H.......  320
Conservation

                                     340                   360                   380                   400
CP2475L_DR-1980       DKELDLKRFS GLVSAENIAE FEKAVNLLRQ TFNERHKILE NSCAKKGGDE EKTPLDRRIE AQRLDRKHIL MEFLNKSTQA  400
LO2018_minor          .......... .......... .......... .......... .......... .......... .......... ..........  400
31479_2015            .......... .......... .......... .......... .......... .......... .......... ..........  400
OR1984                .......... .......... .......... .......... .......... .......... .......... ..........  400
Benin_97/1            .......... .......... .......... .......... .......... .......... .......... ..........  400
15998_2015            .......... .......... .......... .......... .......... .......... .......... ..........  400
Pig/SD/DY-I/2021      .......... .......... .......... .......... .......... .......... .......... ..........  400
Warmbaths             .......... .......... .......... .......... .......... .......... .......... ..........  400
Mkuzi_1979            .......... .......... .......... .......... .......... .......... .......... ..........  400
Tengani_62            .......... .......... .......... .......... .......... .......... .......... ..........  400
ASFV-G                .......... .......... .......... .......... .......... .......... .......... ..........  400
Ulyanovsk_19/WB-5699  .......... .......... .......... .......... .......... .......... .......... ..........  400
HuB20                 .......... .......... .......... .......... .......... .......... .......... ..........  400
Pretorisuskop/96/4    .......... .......... .......... .......... .......... .......... .......... ..........  400
Warthog               .......... .......... .......... .......... .......... .......... .......... ..........  400
K49                   .......... .......... .......... .......... .......... .......... .......... ..........  400
OmLF2                 .......... .......... .......... .......... .......... .......... .......... ..........  400
Malawi_Lil-20/1       .......... .......... .......... .......... .N........ V.....K... .......... ..........  400
Ken05/Tk1             .......... .......... .......... .......... .N........ ......K.M. .......... ..........  400
Kenya_1950            .......... .......... .......... .......... .N.......G ......K.M. .......... ..........  400
Ken.rie1              .......... .......... .......... .......... .N........ ......K.M. .......... ..........  400
Uvira_B53             .......K.. .......... .......... .......... .N........ ......K.M. .......... ..........  400
Ken06.Bus             .......... .......... .......... .......... .N........ ......K.M. .......... ..........  400
N10                   .......... .......... .......... .......... .N........ ......K.M. ........T. ..........  400
Conservation

                                     420                   440                   460                   480
CP2475L_DR-1980       YNDFLENVKK IGIKLVKEIA LTPNITRLRD ALSRINDMGT IALDLSLIGF YTNAAAREER ETFLTQFMLV KNVLEEQSKI  480
LO2018_minor          .......... .......... .......... .......... .......... .......... .......... ..........  480
31479_2015            .......... .......... .......... .......... .......... .......... .......... ..........  480
OR1984                .......... .......... .......... .......... .......... .......... .......... ..........  480
Benin_97/1            .......... .......... .......... .......... .......... .......... .......... ..........  480
15998_2015            .......... .......... .......... .......... .......... .......... .......... ..........  480
Pig/SD/DY-I/2021      .......... .......... .......... .......... .......... .......... .......... ..........  480
Warmbaths             .......... .......... .......... .......... .......... .......... .......... ..........  480
Mkuzi_1979            .......... .......... .......... .......... .......... .......... .......... ..........  480
Tengani_62            .......... .......... .......... .......... .......... .......... ......LT.. ......I..T  480
ASFV-G                .......... .......... .......... .......... .......... .......... ......LT.. ......I..T  480
Ulyanovsk_19/WB-5699  .......... .......... .......... .......... .......... .......... ......LT.. ......I..T  480
HuB20                 .......... .......... .......... .......... .......... .......... ......LT.. ......I..T  480
Pretorisuskop/96/4    .......... .......... .......... .......... .......... .......... .......... .........T  480
Warthog               .......... .......... .......... .......... .......... .......... .......... ..........  480
K49                   .......... .......... .......... .......... .......... .......... ......LT.. ......I..T  480
OmLF2                 .......... .......... .......... .......... .......... .......... .......... ..........  480
Malawi_Lil-20/1       .......... .......... .......... .......... .......... .......... ......LT.. ......I..T  480
Ken05/Tk1             .......... ..M....... ......K... .......... .......... .N........ ....I.LT.. ......LA.T  480
Kenya_1950            .......... ..M....... ......K... .......... .......... .N........ ....I.LT.. ......LA.T  480
Ken.rie1              .......... ..M....... ......K... .......... .......... .N........ ....I.LT.. ......LA.T  480
Uvira_B53             .......... ..M....... ......K... .......... .......... .N........ ....I.LT.. ......LA.T  480
Ken06.Bus             .......... ..M....... ......K... .......... .......... .N........ ....I.LT.. ......LA.T  480
N10                   .......... ..M....... ......K... .......... .......... .N........ ....I.LT.. ......LA.T  480
Conservation
```

CP2475L Alignment

```
                                                                                       500                 520                 540                 560
CP2475L_DR-1980     DPNFKNLYDS CSRLLQIIDF YTDIVQKKYG GGEDCECTRV GGAALTVEEL GLSKAARSQV DLNQAINTFM YYYYVAQIYS 560
LO2018_minor        .......... .......... .......... .......... .......... .......... .......... .......... 560
31479_2015          .......... .......... .......... .......... .......... .......... .......... .......... 560
OR1984              .......... .......... .......... .......... .......... .......... .......... .......... 560
Benin_97/1          .......... .......... .......... .......... .......... .......... .......... .......... 560
15998_2015          .......... .......... .......... .......... .......... .......... .......... .......... 560
Pig/SD/DY-I/2021    .......... .......... .......... .......... .......... .......... .......... .......... 560
Warmbaths           .......... .......... .......... .......... .......... .......... .......... .......... 560
Mkuzi_1979          .......... .......... .......... .......... .......... .......... .......... .......... 560
Tengani_62          .......... .......... .......... .E........ .......... .......... .......... .......... 560
ASFV-G              .......... .......... .......... .E........ .......... .......... .......... .......... 560
Ulyanovsk_19/WB-5699 .......... .......... .......... .E........ .......... .......... .......... .......... 560
HuB20               .......... .......... .......... .E........ .......... .......... .......... .......... 560
Pretorisuskop/96/4  .......... .......... .......... .E........ .......... .......... .......... .......... 560
Warthog             .......... .......... .......... .......... .......... .......... .......... .......... 560
K49                 .......... .......... .......... .......... .......... .......... .......... .......... 560
OmLF2               .......... .......... .......... .......... .......... .......... .......... .......... 560
Malawi_Lil-20/1     .......... .F........ .......... .......... .......... .......... .......... .......... 560
Ken05/Tk1           .......... .F........ .......... ........K. .......... .......... .......... .......... 560
Kenya_1950          .......... .F........ .......... ........K. .......... .......... .......... .......... 560
Ken.rie1            .......... .F........ .......... ........K. .......... .......... .......... .......... 560
Uvira_B53           .......... .F........ .......... ........K. .......... .......... .......... .......... 560
Ken06.Bus           .......... .F........ .......... ........K. .......... .......... .......... .......... 560
N10                 .......... .F........ .......... ........K. .......... .......... .......... .......... 560
Conservation

                                                                                       580                 600                 620                 640
CP2475L_DR-1980     NLTHNKQEFQ SYEENYATIL GDAIAGRLMQ LDTEKNARIN SPAVDLARGH VGPNPGGAQE ADWKAAVSAI ELEYDVKRRF 640
LO2018_minor        .......... .......... .......... .......... .......... .......... .......... .......... 640
31479_2015          .......... .......... .......... .......... .......... ..X....... .......... .......... 640
OR1984              .......... .......... .......... .......... .......... .......... .......... .......... 640
Benin_97/1          .......... .......... .......... .......... .......... .......... .......... .......... 640
15998_2015          .......... .......... .......... .......... .......... .......... .......... .......... 640
Pig/SD/DY-I/2021    .......... .......... .......... .......... .......... .......... .......... .......... 640
Warmbaths           .......... .......... .......... .......... .......... .......... E......... .......... 640
Mkuzi_1979          .......... .......... .......... .......... .......... .......... .......... .......... 640
Tengani_62          .......... .......... .......... .......... .......... .......... E....T.... .......... 640
ASFV-G              .......... .......... .......... .......... .......... .......... V....T.... .......... 640
Ulyanovsk_19/WB-5699 .......... .......... .......... .......... .......... .......... V....T.... .......... 640
HuB20               .......... .......... .......... .......... .......... .......... V....T.... .......... 640
Pretorisuskop/96/4  .......... .......... .......... .......... .......... .......... V......... .......... 640
Warthog             .......... .......... .......... .......... .......... .......... E....T.... .......... 640
K49                 .......... .......... .......... .......... .......... .......... .......... .......... 640
OmLF2               .......... .......... .......... .......... .......... .......... V......... .......... 640
Malawi_Lil-20/1     .......... .......... .......... .......... .......... .......... V......... .......... 640
Ken05/Tk1           .......... .......... .......... .......... .......... .......... V....TI... .......... 640
Kenya_1950          .......... .......... .......... .......... .......... .......... V....TI... .......... 640
Ken.rie1            .......... .......... .......... .......... .......... .......... V....TI... .......... 640
Uvira_B53           .......... .......... .......... .......... .......... .......... I....TI... .......... 640
Ken06.Bus           .......... .......... .......... .......... .......... .......... V....TI... .......... 640
N10                 .......... .......... .......... .......... .......... .......... V....TI... .......... 640
Conservation

                                                                                       660                 680                 700                 720
CP2475L_DR-1980     YRALEGLDLY LKNITKTFVN NIDSIQTVQQ MLDGVRIIGR WFTEATGDTL AQVFESFPTS AGNDSNVFTD NAPAGHYYEK 720
LO2018_minor        .......... .......... .......... .......... .......... .......... .......... .......... 720
31479_2015          .......... .......... .......... .......... .......... .......... .......... .......... 720
OR1984              .......... .......... .......... .......... .......... .......... .......... .......... 720
Benin_97/1          .......... .......... .......... .......... .......... .......... .......... .......... 720
15998_2015          .......... .......... .......... .......... .......... .......... .......... .......... 720
Pig/SD/DY-I/2021    .......... .......... .......... .......... .......... .......... .......... .......... 720
Warmbaths           .......... .......... .......... .......... .......... .......... .......... .......... 720
Mkuzi_1979          .......... .......... .......... .......... .......... .......... .......... .......... 720
Tengani_62          .......... .......... .......... .......... .......... .......... T......... .......... 720
ASFV-G              .......... .......... .......... .......... ....T..... .......... T......... .......... 720
Ulyanovsk_19/WB-5699 .......... .......... .......... .......... ....T..... .......... T......... .......... 720
HuB20               .......... .......... .......... .......... ....T..... .......... T......... .......... 720
Pretorisuskop/96/4  .......... .......... .......... .......... .......... .......... .......... .......... 720
Warthog             .......... .......... .......... .......... .......... .......... .......... .......... 720
K49                 .......... .......... .......... .......... .......... .......... .......... .......... 720
OmLF2               .......... .......... .......... .......... .......... .......... .......... .......... 720
Malawi_Lil-20/1     .......... .......... .......... .......... .......... .......... .......... .......... 720
Ken05/Tk1           .......... .......... .......... .......... .......... .......... .......... .......... 720
Kenya_1950          .......... .......... .......... .......... .......... .......... .......... .......... 720
Ken.rie1            .......... .......... .......... .......... .......... .......... .......... .......... 720
Uvira_B53           .......... .......... .......... .......... .......... .......... .......... .......... 720
Ken06.Bus           .......... .......... .......... .......... .......... .......... .......... .......... 720
N10                 .......... .......... .......... .......... .......... .......... .......... .......... 720
Conservation
```

CP2475L Alignment

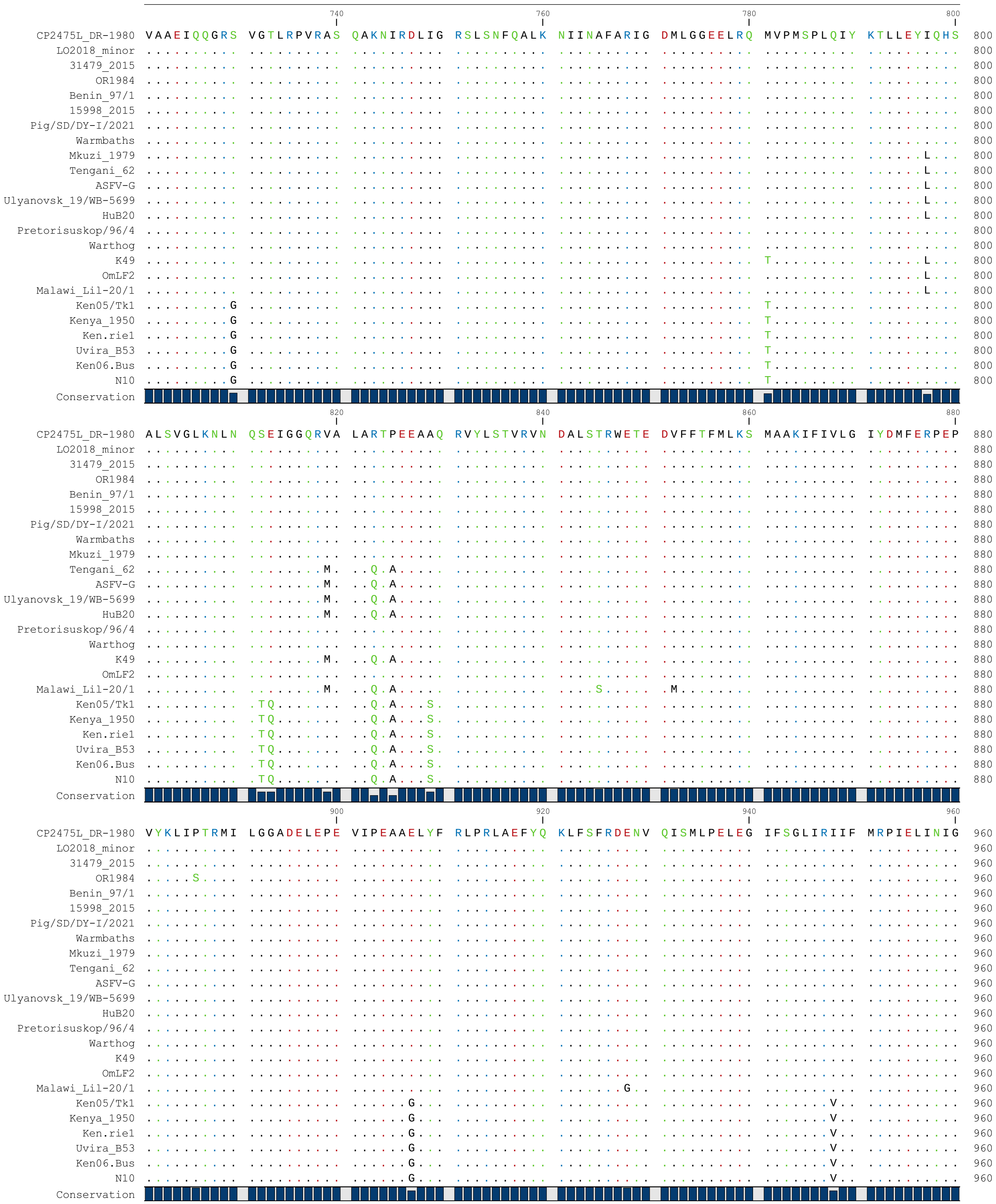

CP2475L Alignment

```
                                       980                 1,000                 1,020                 1,040
CP2475L_DR-1980       DYSETEIRQL IKEINVIYQH FNLEYGEQEA TKKALIHFVN EINRRFGVIT RTEWEKFQRI VQEARTMNDF GMMNQTNYSI 1040
LO2018_minor          .......... .......... .......... .......... .......... .......... .......... .......... 1040
31479_2015            .......... .......... .......... .......... .......... .......... .......... .......... 1040
OR1984                .......... .......... .......... .......... .......... .......... .......... .......... 1040
Benin_97/1            .......... .......... .......... .......... .......... .......... .......... .......... 1040
15998_2015            .......... .......... .......... .......... .......... .......... .......... .......... 1040
Pig/SD/DY-I/2021      .......... .......... .......... .......... .......... .......... .......... .......... 1040
Warmbaths             .......... .......... .......... .......... .......... .......... .......... .......... 1040
Mkuzi_1979            .......... .......... .......... .......... .......... .......... .......... .......... 1040
Tengani_62            .......... .......... .......... .......... .......... .......... .......... .......... 1040
ASFV-G                .......... .......... .......... .......... .......... .......... .......... .......... 1040
Ulyanovsk_19/WB-5699  .......... .......... .......... .......... .......... .......... .......... .......... 1040
HuB20                 .......... .......... .......... .......... .......... .......... .......... .......... 1040
Pretorisuskop/96/4    .......... .......... .......... .......... .......... .......... .......... .......... 1040
Warthog               .......... .......... .......... .......... .......... .......... .......... .......... 1040
K49                   .......... .......... .......... .......... .......... .......... .......... .......... 1040
OmLF2                 .......... .......... .......... .......... .......... .......... .......... .......... 1040
Malawi_Lil-20/1       .......... .......... .......... .......... .......... .......... .......... .......... 1040
Ken05/Tk1             .......... .......... .......... V......... .......... .......... .......... .......... 1040
Kenya_1950            .......... .......... .......... V......... .......... .......... .......... .......... 1040
Ken.rie1              .......... .......... .......... V......... .......... .......... .......... .......... 1040
Uvira_B53             .......... .......... .......... V......... .......... .......... .......... .......... 1040
Ken06.Bus             .......... .......... .......... V......... .......... .......... .......... .......... 1040
N10                   .......... .......... .......... V......... .......... .......... .......... .......... 1040
Conservation

                                      1,060                1,080                 1,100                 1,120
CP2475L_DR-1980       LPDEDGYTQS SQLLPSDRFI SPSTQPTPKW RPALYNIDSV DVQTGMLQPN SQWDLVQKFR KQLSEMFEDP SLQQELGKVS 1120
LO2018_minor          .......... .......... .......... .......... .......... .......... .......... .......... 1120
31479_2015            .......... .......... .......... .......... .......... .......... .......... .......... 1120
OR1984                .......... .......... .......... .......... .......... .......... .......... .......... 1120
Benin_97/1            .......... .......... .......... .......... .......... .......... .......... .......... 1120
15998_2015            .......... .......... .......... .......... .......... .......... .......... .......... 1120
Pig/SD/DY-I/2021      .......... .......... .......... .......... .......... .......... .......... .......... 1120
Warmbaths             .......... .......... ...S...... .......... .......... .......... .......... .......... 1120
Mkuzi_1979            .......I.. .......... ......S... .......... .......... .......... .......... .......... 1120
Tengani_62            .......... .......... .......... .......... .......... .......... .......... ........I. 1120
ASFV-G                .......... .......... .......... .......... .......... .......... .......... ........I. 1120
Ulyanovsk_19/WB-5699  .......... .......... .......... .......... .......... .......... .......... ........I. 1120
HuB20                 .......... .......... .......... .......... .......... .......... .......... ........I. 1120
Pretorisuskop/96/4    .......... .......... ...S...... .......... .......... .......... .......... .......... 1120
Warthog               .......... .......... ...S...... .......... .......... .......... .......... .......... 1120
K49                   .......... .......... .......... .......... .......... .......... .......... ........I. 1120
OmLF2                 .......I.. .......... ......S... .......... .......... .......... .......... .......... 1120
Malawi_Lil-20/1       .......... .......... G..S...... .......... .......... .......... .......... ........I. 1120
Ken05/Tk1             .......... .......... G..S...... .......... .......... .......... .......... .......... 1120
Kenya_1950            .......... .......... G..S...... .......... .......... .......... .......... .......... 1120
Ken.rie1              .......... .......... G..S...... .......... .......... .......... .......... .......... 1120
Uvira_B53             .......... .......... G..S...... .......... .......... .......... .......... .......... 1120
Ken06.Bus             .......... .......... G..S...... .......... .......... .......... .......... .......... 1120
N10                   .......... .......... G..S...... .......... .......... .......... .......... .......... 1120
Conservation

                                      1,140                1,160                 1,180                 1,200
CP2475L_DR-1980       YQELIRQAIN ELKKEHTDKI QIVSKLIQGS ESLADTDVNK IFLFHETVIT GLNLLSAIYV LLNNFRNNIK GLDLDTIQKS 1200
LO2018_minor          .......... .......... .......... .......... .......... .......... .......... .......... 1200
31479_2015            .......... .......... .......... .......... .......... .......... .......... .......... 1200
OR1984                .......... .......... .......... .......... .......... .......... .......... .......... 1200
Benin_97/1            .......... .......... .......... .......... .......... .......... .......... .......... 1200
15998_2015            .......... .......... .......... .......... .......... .......... .......... .......... 1200
Pig/SD/DY-I/2021      .......... .......... .......... .......... .......... .......... .......... .......... 1200
Warmbaths             .......... ....D..... .......... .......... .......... .......... .......... .......... 1200
Mkuzi_1979            .......... ....D..... .......... .......... .......... .......... .......... .......... 1200
Tengani_62            .......... .......... .......... .......... .......... .......... .......... .......... 1200
ASFV-G                .......... .......... .......... .......... .......... .......... .......... .......... 1200
Ulyanovsk_19/WB-5699  .......... .......... .......... .......... .......... .......... .......... .......... 1200
HuB20                 .......... .......... .......... .......... .......... .......... .......... .......... 1200
Pretorisuskop/96/4    .......... ....D..... .......... .......... .......... .......... .......... .......... 1200
Warthog               .......... ....D..... .......... .......... .......... .......... .......... .......... 1200
K49                   .......... .......... .......... .......... .......... .......... .......... .......... 1200
OmLF2                 .......... .......... .......... .......... .......... .......... .......... .......... 1200
Malawi_Lil-20/1       .......... .......... .......... .......... .......... .......... .......... .......... 1200
Ken05/Tk1             .....Q..T. .......... .......... .......... .......... .......... ...T...... A......... 1200
Kenya_1950            .....Q..T. .......... .......... .......... .......... .......... ...T...... A......... 1200
Ken.rie1              .....Q..T. .......... .......... .......... .......... .......... ...T...... A......... 1200
Uvira_B53             .....Q..T. .......... .......... .......... .......... .......... ...T...... A......... 1200
Ken06.Bus             .....Q..T. .......... .......... .......... .......... .......... ...T...... A....M.... 1200
N10                   .....Q..T. .......... .......... .......... .......... .......... ...T...... A....M.... 1200
Conservation
```

CP2475L Alignment

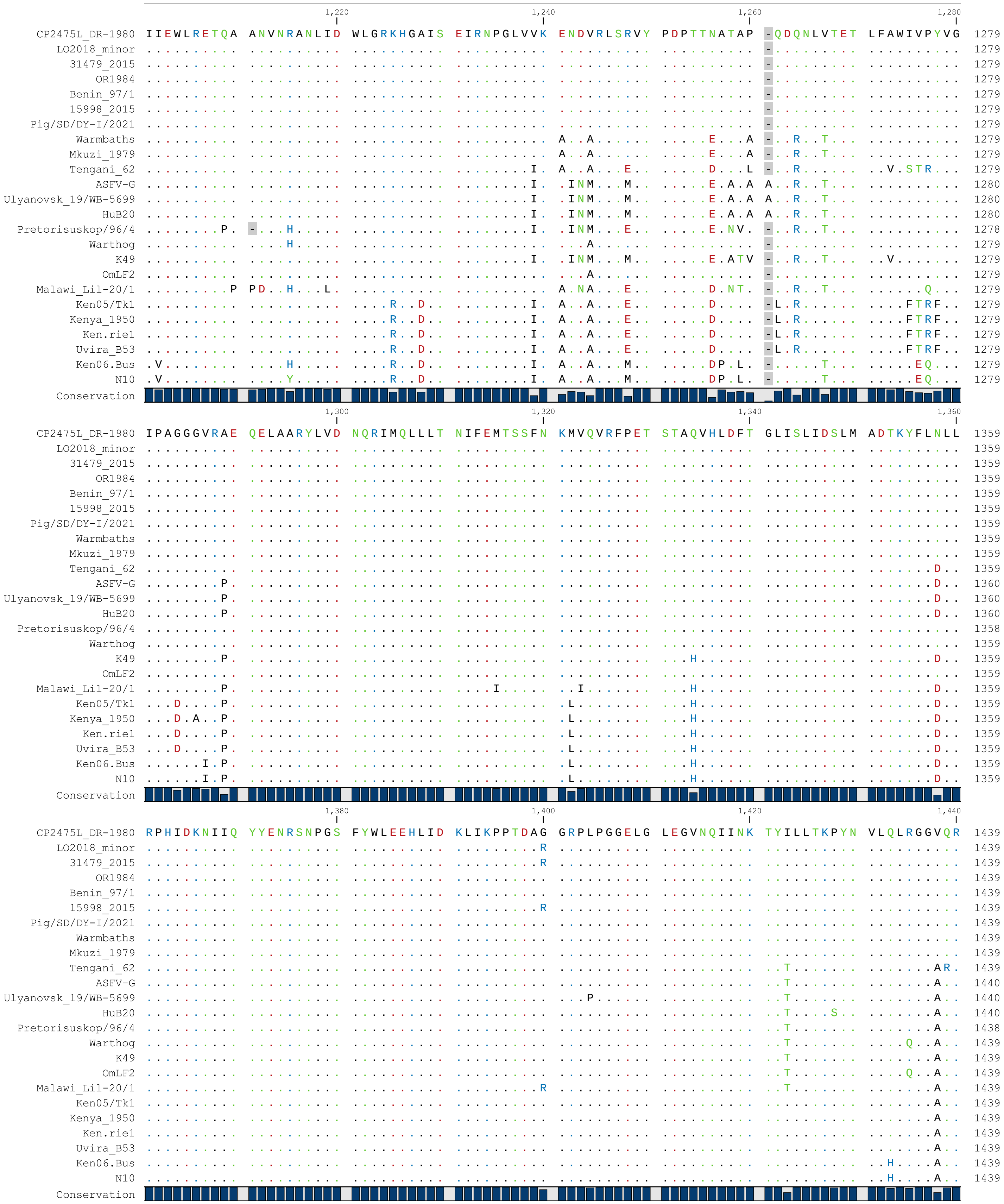

CP2475L Alignment

```
                                                  1,460                                              1,480                                              1,500                                              1,520
CP2475L_DR-1980       RDAANIQINN NPQPSERFEQ YGRVFSRLVF YDALENNSGL RVEQVVLGDF RLSNLIRTNN AQEENTLSYW DNMAP--RTY  1517
LO2018_minor          .......... .......... .......... .......... .......... .......... .......... .....--...  1517
31479_2015            .......... .......... .......... .......... .......... .......... .......... .....--...  1517
OR1984                .......... .......... .......... .......... .......... .......... .......... .....--...  1517
Benin_97/1            .......... .......... .......... .......... .......... .......... .......... .....--...  1517
15998_2015            .......... .......... .......... .......... .......... .......... .......... .....--...  1517
Pig/SD/DY-I/2021      .......... .......... .......... .......... .......... .......... .......... .....--...  1517
Warmbaths             .......... .......... .......... .......... .......... .......... .......... .....--...  1517
Mkuzi_1979            .......... .......... .......... .......... .......... .......... .......... .....--...  1517
Tengani_62            G......... .......... ........I. .......... .....A.... .......... .......... .....--...  1517
ASFV-G                .......... ...S...... .......... .......... .....A.... .......... .......... ..I.L--...  1518
Ulyanovsk_19/WB-5699  .......... ...S...... .......... .......... .....A.... .......... .......... ..I.L--...  1518
HuB20                 .......... ...S...... .......... .......... .....A.... .......... .......... ..I.L--...  1518
Pretorisuskop/96/4    .......... .......... .......... .......... .......... .......... .....A.... ..I.L--...  1516
Warthog               .......... .......... .......... .......... .......... .......... .....A.... ..I.L--...  1517
K49                   .......... ...S...... .......... .......... .....A.... .......... .....A.... ..I.L--...  1517
OmLF2                 .......... .......... .......... .......... .......... .......... .....A.... ..I.L--...  1517
Malawi_Lil-20/1       .......... ...S...... ......K... .......... .....A.... .......... .......... ..I.L--...  1517
Ken05/Tk1             GN........ ..EL...Y.. .......... ....IE.... .....A.... .....V.... .....A..F. TAV..--.A.  1517
Kenya_1950            GN........ ..EF...Y.. .......... ....IE.... .....A.... .......... .....A..F. TAV..--.A.  1517
Ken.rie1              GN........ ..EL...Y.. .......... ....IE.... .....A.... .....V.... .....A..F. TAV..--.A.  1517
Uvira_B53             GN........ ..EH...Y.. .......... ....IE.... .....A.... .....V.... .....A..F. TAV..--.A.  1517
Ken06.Bus             .......... ..EL...Y.. .......... ....D..... .....A.... .......... .....A..F. TAV..AP.A.  1519
N10                   .......... ..EL...Y.. .......... ....D..... .....A.... .......... .....A..F. TAV..AP.A.  1519
Conservation

                                                  1,540                                              1,560                                              1,580                                              1,600
CP2475L_DR-1980       ANVNDAANNL RRYRLYGSDY GIQNNRSMMM VFNQLVASYI ARFYDAPSGK IYLNLINAFA NGNFSQAVME LGYTHPDLAR  1597
LO2018_minor          .......... .......... .......... .......... .......... .......... .......... ..........  1597
31479_2015            .......... .......... .......... .......... .......... .......... .......... ..........  1597
OR1984                .......... .......... .......... .......... .......... .......... .......... ..........  1597
Benin_97/1            .......... .......... .......... .......... .......... .......... .......... ..........  1597
15998_2015            .......... .......... .......... .......... .......... .......... .......... ..........  1597
Pig/SD/DY-I/2021      .......... .......... .......... .......... .......... .......... .......... ..........  1597
Warmbaths             .......... .......... .......... .......... .......... .......... .......... ..........  1597
Mkuzi_1979            .......... .......... .......... .......... .......... .......... .......... ..........  1597
Tengani_62            ..I....... .......... ..R....... .......... .......... .......... .......... M..A......  1597
ASFV-G                .......... .......... .......... .....I.... T......... .......... .......... M..A......  1598
Ulyanovsk_19/WB-5699  .......... .......... .......... .....I.... T......... .......... .......... M..A......  1598
HuB20                 .......... .......... .......... .....I.... T......... .......... .......... M..A......  1598
Pretorisuskop/96/4    .......... .........H .......... .......... .......... .......... .......... M..A......  1596
Warthog               .......... .........H .......... .......... .......... .......... .......... M..A......  1597
K49                   .......... .......... ..R....... .......... .......... .......... .......... ..........  1597
OmLF2                 .......... .........Q .......... .......... .......... .......... .......... M..A......  1597
Malawi_Lil-20/1       .......... .......... .......... .....I.... T......... .......... .......... M..A......  1597
Ken05/Tk1             .......... .......... ..R....... .......... .......... .......... .......... ...A......  1597
Kenya_1950            .......... .......... ..R....... .......... .......... .......T.. .......... ...A......  1597
Ken.rie1              .......... .......... ..R....... .......... .......... .......... .......... ...A......  1597
Uvira_B53             .......... .......... ..R....... .......... .......... .......T.. .......... ...A......  1597
Ken06.Bus             ....N..... .......... ..R....... .......... .......... .......... .......... ...A......  1599
N10                   ....N..... .......... ..R....... .......... .......... .......... .......... ...A......  1599
Conservation

                                                  1,620                                              1,640                                              1,660                                              1,680
CP2475L_DR-1980       DNIAFGHRGD PTEQSVLLLS LGLMLQRLIK DTNRQGLSQH LISTLTEIPI YLKENYRANL PLFNKMFNIL ISQGELLKQF  1677
LO2018_minor          .......... .......... .......... .......... .......... .......... .......... ..........  1677
31479_2015            .......... .......... .......... .......... .......... .......... .......... ..........  1677
OR1984                .......... .......... .......... .......... .......... .......... .......... ..........  1677
Benin_97/1            .......... .......... .......... .......... .......... .......... .......... ..........  1677
15998_2015            .......... .......... .......... .......... .......... .......... .......... ..........  1677
Pig/SD/DY-I/2021      .......... .......... .......... .......... .......... .......... .......... ..........  1677
Warmbaths             .......... .......... .......... .......... .......... .......... .......... ..........  1677
Mkuzi_1979            .......... .......... .......... .......... .......... .......... .......... ..........  1677
Tengani_62            N......... .......... .......... .......... .......... .......... .......... ..........  1677
ASFV-G                N.NV...... .......... ...I...... .......... .......... .......... .......... ..........  1678
Ulyanovsk_19/WB-5699  N.NV...... .......... ...I...... .......... .......... .......... .......... ..........  1678
HuB20                 N.NV...... .......... ...I...... .......... .........V .......... .......... ..........  1678
Pretorisuskop/96/4    N.N....... .......... ...I...... .......... .......... .......... .......... ..........  1676
Warthog               N.N....... .......... ...I...... .......... .......... .......... .......... ..........  1677
K49                   .......... .......... .......... .......... .......... .......... .......... ..........  1677
OmLF2                 N.N....... .......... ...I...... .......... .......... .......... .......... ..........  1677
Malawi_Lil-20/1       N.N....... .......... ...I...... .......... .......... .......... .......... ..........  1677
Ken05/Tk1             ..T....... .......... .......... .......... .......... .......... .......... ..........  1677
Kenya_1950            ..T....... .......... .......... .......... .......... .......... .......... ..........  1677
Ken.rie1              ..T....... .......... .......... .......... .......... .......... .......... ..........  1677
Uvira_B53             ..T....... .......... .......... .......... .......... .......... .......... ..........  1677
Ken06.Bus             .......... .......... .......... .......... .......... .......... ..Y....... ..........  1679
N10                   .......... .......... .......... .......... .......... .......... ..Y....... ..........  1679
Conservation
```

CP2475L Alignment

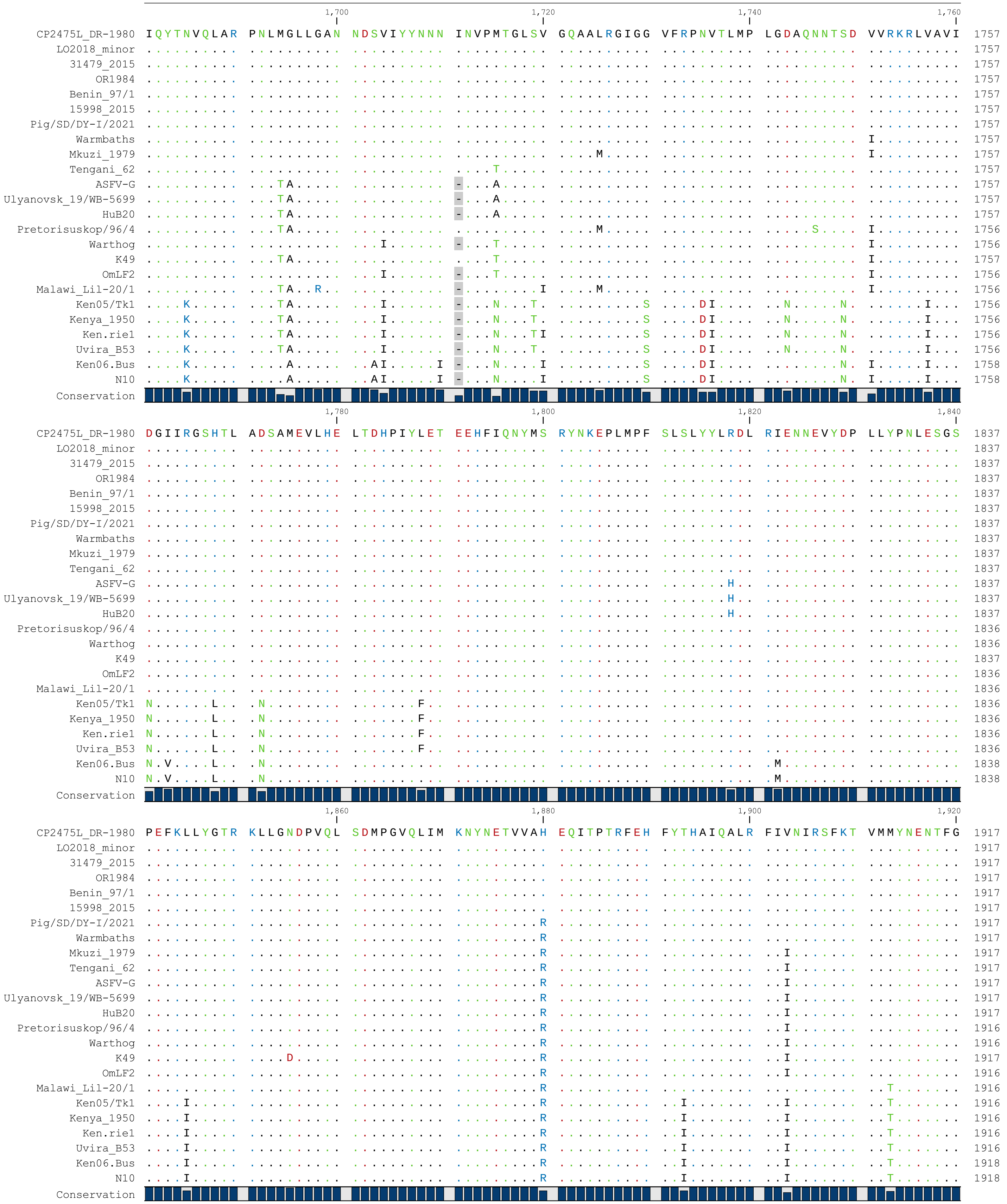

CP2475L Alignment

1,940 · 1,960 · 1,980 · 2,000

CP2475L_DR-1980	GVNLISENRD DKPIITAGIG MNAVYSLRKT LQDVISFVES SYQEEQINHI HKIVSPKGQT RTLGSNRERE RIFNLFDMNI	1997
LO2018_minor	....	1997
31479_2015	....	1997
OR1984	....	1997
Benin_97/1	....	1997
15998_2015	....	1997
Pig/SD/DY-I/2021	....	1997
Warmbaths	....	1997
Mkuzi_1979	....	1997
Tengani_62	....	1997
ASFV-G	....	1997
Ulyanovsk_19/WB-5699	....	1997
HuB20	....	1997
Pretorisuskop/96/4	....	1996
Warthog	....	1996
K49	....	1997
OmLF2	....	1996
Malawi_Lil-20/1	.......D.N ....N.	1996
Ken05/Tk1	.......D.. ......E... ....N. ......RS.. .S........	1996
Kenya_1950	.....G.D.. ......E... ....N. ......RS.. .S........	1996
Ken.rie1	.....G.D.. ......E... ....N. ......RS.. .S........	1996
Uvira_B53	.....G.D.. ......E... ....N. ......RS.. .S........	1996
Ken06.Bus	....N. ....S.. .S........	1998
N10	....N. ....S.. .S........	1998
Conservation		

2,020 · 2,040 · 2,060 · 2,080

CP2475L_DR-1980	IPINVNALMR SIPLANIYNY DYSFEEIACL MYGISAEKVR SLNTAAAQPD IAEVLNIPNR PPMNTREFML KLLINPYVSV	2077
LO2018_minor	....P...	2077
31479_2015	....P...	2077
OR1984	....P...	2077
Benin_97/1	....P...	2077
15998_2015	....P...	2077
Pig/SD/DY-I/2021	....P...	2077
Warmbaths	....P...	2077
Mkuzi_1979	....P...	2077
Tengani_62	....P...	2077
ASFV-G	....D.T.P... V......... ..I.......	2077
Ulyanovsk_19/WB-5699	....D.T.P... V......... ..I.......	2077
HuB20	....D.T.P... V......... ..I.......	2077
Pretorisuskop/96/4	....P...	2076
Warthog	....D.T.P... V......... ..I.......	2076
K49	....P... V.........	2077
OmLF2	....P... V.........	2076
Malawi_Lil-20/1	....P... V......... ..I.......	2076
Ken05/Tk1	M......... ....D...P... V.Q....... ....T.	2076
Kenya_1950	M......... ....D...P... V.Q....... ....T.	2076
Ken.rie1	M......... ....D...P... V.Q....... ....T.	2076
Uvira_B53	M......... ....D...P... V.Q....... ....T.	2076
Ken06.Bus	M......... ....D.G.P... V.V....... ....T.	2078
N10	M......... ....D.G.P... V.V....... ....T.	2078
Conservation		

2,100 · 2,120 · 2,140 · 2,160

CP2475L_DR-1980	SITQYGNELM SKGSAGYMSR IFRGDNALNM GRPKFLSDQI FNKVLFGSLY PTQFDYDEAG PGLAAGIQRG REQWGQPLSE	2157
LO2018_minor	....	2157
31479_2015	....	2157
OR1984	....	2157
Benin_97/1	....	2157
15998_2015	....	2157
Pig/SD/DY-I/2021	....	2157
Warmbaths	....	2157
Mkuzi_1979	....	2157
Tengani_62	....	2157
ASFV-G	.........L ...N...... .... .S........ ..R..H.M.I	2157
Ulyanovsk_19/WB-5699	.........L ...N...... .... .S........ ..R..H.M.I	2157
HuB20	.........L ...N...... .... .S........ ..R..H.M.I	2157
Pretorisuskop/96/4	.........L ...N...... .... .S........ ..R..H.M.I	2156
Warthog	.........L ...N...... ....R..H.M.I	2156
K49	.........L ...N...... ....R..H.M.I	2157
OmLF2	.........L ...N...... ....R..H.M.I	2156
Malawi_Lil-20/1	.........L ...N...... .... .S........ ..R..H.M.I	2156
Ken05/Tk1	.........L FR.NT..... ....F.D	2156
Kenya_1950	.........L FR.N...... ....D	2156
Ken.rie1	.........L FR.NT..... ....F.D	2156
Uvira_B53	.........L FR.NT..... ....F.D	2156
Ken06.Bus	..S......L .R.N...... ....R..H.M.I	2158
N10	..S......L .R.N...... ....R..H.M.I	2158
Conservation		

CP2475L Alignment

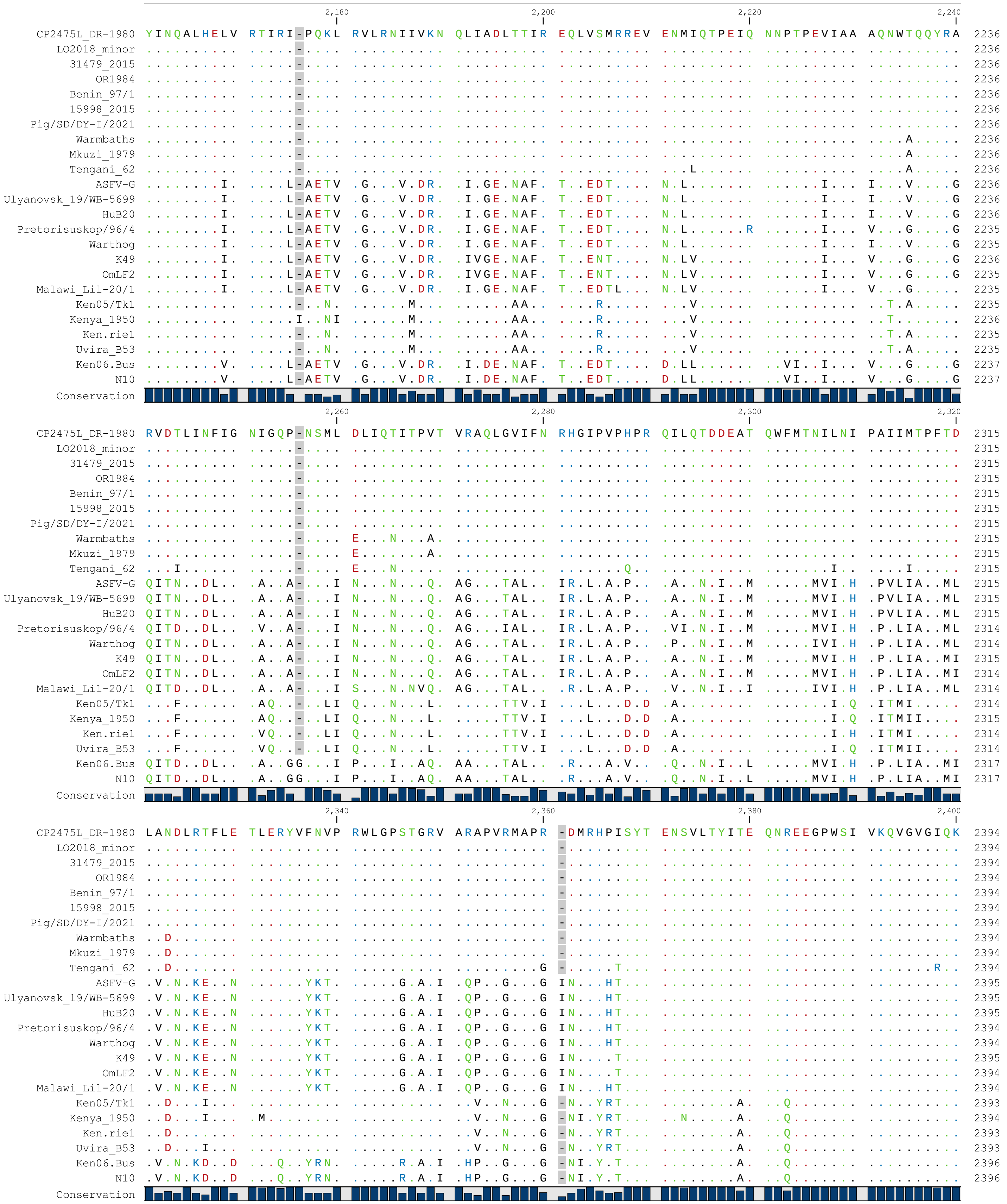

CP2475L Alignment

```
                                   2,420      2,440      2,460      2,480
CP2475L_DR-1980        PTLVQIGKDR FDTRLIRNLI FITNIQRLLR LRLNLELSQF RNVLVSPDHI INPSITEYGF SITGPSETFS DKQYDSDIRI 2474
LO2018_minor           .......... .......... .......... .......... .......... .......... .......... .......... 2474
31479_2015             .......... .......... .......... .......... .......... .......... .......... .......... 2474
OR1984                 .......... .......... .......... .......... .......... .......... .......... .......... 2474
Benin_97/1             .......... .......... .......... .......... .......... .......... .......... .......... 2474
15998_2015             .......... .......... .......... .......... .......... .......... .......... .......... 2474
Pig/SD/DY-I/2021       .......... .......... .......... .......... .......... .......... .......... .......... 2474
Warmbaths              .......... .......... .......... .......... .......... .......... .......... .......... 2474
Mkuzi_1979             .......... .......... .......... .......... .......... .......... .......... .......... 2474
Tengani_62             .......... .......... .......... .......... .......... .......... .......... .......... 2474
ASFV-G                 .......... .......... .......... .......... .......... .......... .......... .......... 2475
Ulyanovsk_19/WB-5699   .......... .......... .......... .......... .......... .......... .......... .......... 2475
HuB20                  .......... .......... .......... .......... .......... .......... .......... .......... 2475
Pretorisuskop/96/4     .......... .......... .......... .......... .......... .......... .......... .......... 2474
Warthog                ....H..... .......... .......... .......... .......... .......... .......... .......... 2474
K49                    .......... .......... .......... .......... .......... .......... .......... .......... 2475
OmLF2                  .......... .......... .......... .......... .......... .......... .......... .......... 2474
Malawi_Lil-20/1        .......... .......... ....V..... .......... .......... .......... .......... .......... 2474
Ken05/Tk1              .A.I...... .......... .......... .......... .......... .......... .......... .......... 2473
Kenya_1950             .A.I...... .......... .......... .......... .......N.. .......... .......... .......... 2474
Ken.rie1               .A.I...... .......... .......... .......... .......... .......... .......... .......... 2473
Uvira_B53              .A.I...... .......... .......... .......... .......... .......... .......... .......... 2473
Ken06.Bus              .A.I...... .......... .......... .......... .......... .......... .......... .......... 2476
N10                    .A.I...... .......... .......... .......... .......... .......... .......... .......... 2476
Conservation

CP2475L_DR-1980        L  2475
LO2018_minor           .  2475
31479_2015             .  2475
OR1984                 .  2475
Benin_97/1             .  2475
15998_2015             .  2475
Pig/SD/DY-I/2021       .  2475
Warmbaths              .  2475
Mkuzi_1979             .  2475
Tengani_62             .  2475
ASFV-G                 .  2476
Ulyanovsk_19/WB-5699   .  2476
HuB20                  .  2476
Pretorisuskop/96/4     .  2475
Warthog                .  2475
K49                    .  2476
OmLF2                  .  2475
Malawi_Lil-20/1        .  2475
Ken05/Tk1              .  2474
Kenya_1950             .  2475
Ken.rie1               .  2474
Uvira_B53              .  2474
Ken06.Bus              .  2477
N10                    .  2477
Conservation
```

G1211R Alignment

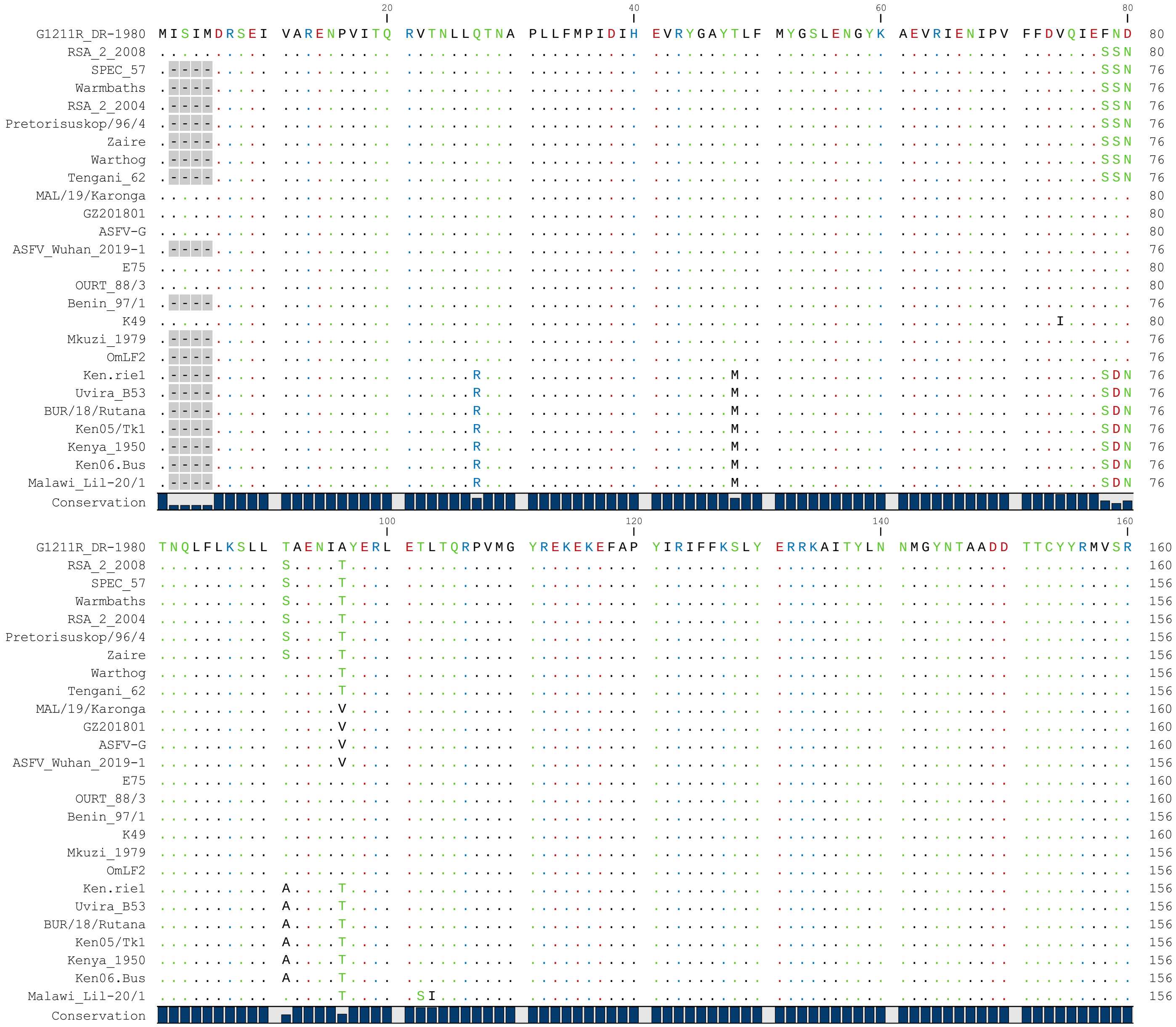

G1211R Alignment

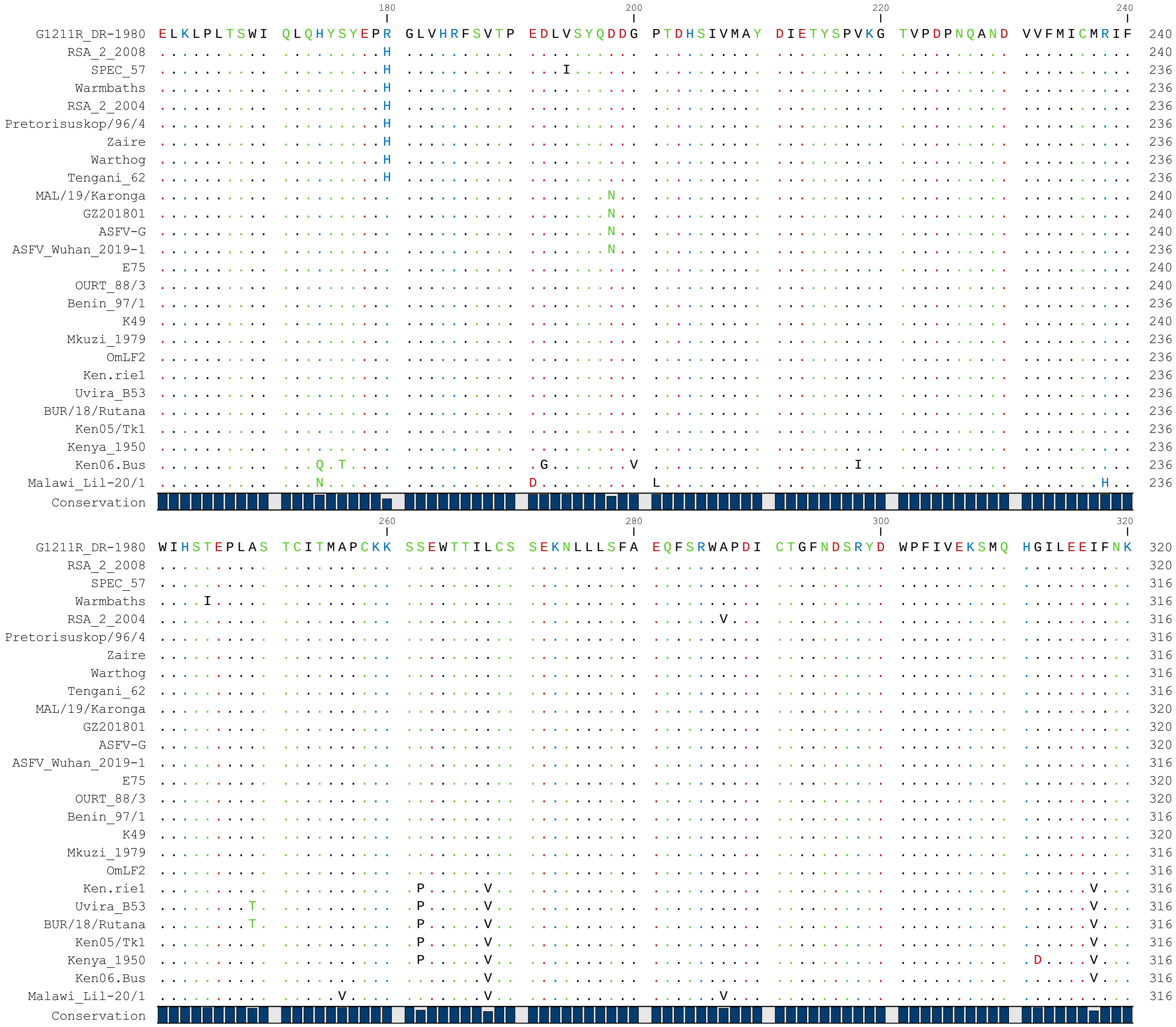

G1211R Alignment

```
                                                           340                  360                  380                  400
                                                             |                    |                    |                    |
G1211R_DR-1980        MSLFWHQKLD TILKCYYVKE KRVKISAEKS IISSFLHTPG CLPIDVRNMC MQLYPKAEKT SLKAFLENCG LDSKVDLPYH  400
RSA_2_2008            .......... .......... .......... .......... .......... .......... .......... ..........  400
SPEC_57               .......... .......... .......... .......... .......... .......... .......... ..........  396
Warmbaths             .......... .......... .......... .......... .......... .......... .......... ..........  396
RSA_2_2004            .......... .......... .......... .......... .......... .......... .......... ..........  396
Pretorisuskop/96/4    .......... .......... .......... .......... .......... .......... .......... ..........  396
Zaire                 .......... .......... .......... .......... .......... .......... .......... ..........  396
Warthog               .......... .......... .......... .......... .......... .......... .......... ..........  396
Tengani_62            .......... .......... .......... .......... .......... .......... .......... ..........  396
MAL/19/Karonga        .......... .......... .......... .......... .......... .......... .......... ..........  400
GZ201801              .......... .......... .......... .......... .......... .......... .......... ..........  400
ASFV-G                .......... .......... .......... .......... .......... .......... .......... ..........  400
ASFV_Wuhan_2019-1     .......... .......... .......... .......... .......... .......... .......... ..........  396
E75                   .......... .......... .......... .......... .......... .......... .......... ..........  400
OURT_88/3             .......... .......... .......... .......... .......... .......... .......... ..........  400
Benin_97/1            .......... .......... .......... .......... .......... .......... .......... ..........  396
K49                   .......... .......... .......... .......... .......... .......... .......... ..........  400
Mkuzi_1979            .......... .......... .......... .......... .......... .......... .......... ..........  396
OmLF2                 .......... .......... .......... ........I. .......... .......... .......... ..........  396
Ken.rie1              .....P.... .......... .......... .......... .......... .......... .......... ..........  396
Uvira_B53             .....P.... .......... .......... .......... .......... .......... ...S...... ..........  396
BUR/18/Rutana         .....P.... .......... .......... .......... .......... .......... ...S...... ..........  396
Ken05/Tk1             .....P.... .......... .......... .......... .......... .......... .......... ..........  396
Kenya_1950            .....P.... .......... .......... .......... ...M...... .......... .......... ..........  396
Ken06.Bus             .....P.... .......... .......... .......... .......... .......... .......... ....I.....  396
Malawi_Lil-20/1       .....N.... .......... .......... .......... ...M...... ..I....... .......... ..........  396
Conservation

                                                           420                  440                  460                  480
                                                             |                    |                    |                    |
G1211R_DR-1980        LMWKYYETRD SEKMADVAYY CIIDAQRCQD LLVRHNVIPD RREVGILSYT SLYDCIYYAG GHKVCNMLIA YAIHDEYGRI  480
RSA_2_2008            .......... .......... .......... .......... .......... .......... .......... ..........  480
SPEC_57               .......... .......... .......... .......... .......... .......... .......... ..........  476
Warmbaths             .......... .......... .......... .......... .......... .......... .......... ..........  476
RSA_2_2004            .......... .......... .......... .......... .......... .......... .......... ..........  476
Pretorisuskop/96/4    .......... .......... .......... .......... .......... .......... .......... ..........  476
Zaire                 .......... .......... .......... .......... .......... .......... .......... ..........  476
Warthog               .......... .......... .......... .......... .......... .......... .......... ..........  476
Tengani_62            .........N .......... .......... .......... .......... .......... .......... ..........  476
MAL/19/Karonga        .......... ...I...... .......... .......... .......... .......... .......... ..........  480
GZ201801              .......... ...I...... .......... .......... .......... .......... .......... ..........  480
ASFV-G                .......... ...I...... .......... .......... .......... .......... .......... ..........  480
ASFV_Wuhan_2019-1     .......... ...I...... .......... .......... .......... .......... .......... ..........  476
E75                   .......... .......... .......... .......... .......... .......... .......... ..........  480
OURT_88/3             .......... .......... .......... .......... .......... .......... .......... ..........  480
Benin_97/1            .......... .......... .......... .......... .......... .......... .......... ..........  476
K49                   .......... .......... .......... .......... .......... .......... .......... ..........  480
Mkuzi_1979            .......... .......... .......... .......... .......... .......... .......... ..........  476
OmLF2                 .......... .......... .......... .......... .......... .......... .......... ..........  476
Ken.rie1              .......... .......... .......... .......... .......... .......... .......... ..........  476
Uvira_B53             .......... .......... .......... .......... .......... .......... .......... ..........  476
BUR/18/Rutana         .......... .......... .......... .......... .......... .......... .......... ..........  476
Ken05/Tk1             .......... .......... .......... .......... .......... .......... .......... ..........  476
Kenya_1950            .......... .......... .......... .......... .......... .......... .......... ..........  476
Ken06.Bus             .......... .......... .......... ........A. .......... .......... .......... ..........  476
Malawi_Lil-20/1       .......... .......... .......... .......... .......... .......... .......... ..........  476
Conservation
```

G1211R Alignment

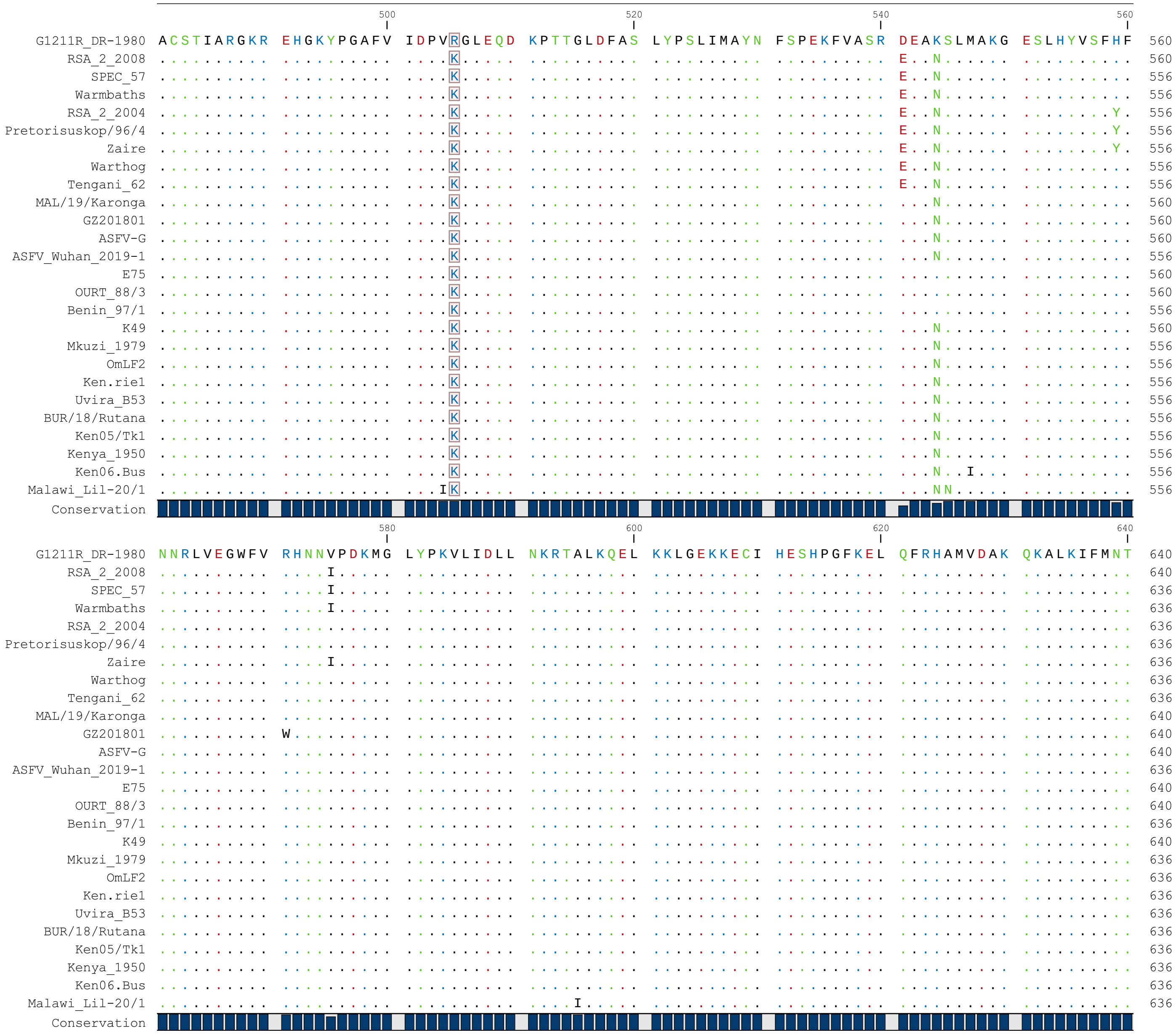

G1211R Alignment

```
                           660                 680                 700                 720
G1211R_DR-1980      FYGEAGNNLS PFFLLPLAGG VTSSGQYNLK LVYNFVINKG YGIKYGDTDS LYITCPDSLY TEVTDAYLNS QKTIKHYEQL 720
RSA_2_2008          .......... .......... .......... .......... .......... .......... .......... .......... 720
SPEC_57             .......... .......... .......... .......... .......... .......... .......... .......... 716
Warmbaths           .......... .......... .......... .......... .......... .......... .......... .......... 716
RSA_2_2004          .......... .......... .......... .......... .......... .......... .......... .......... 716
Pretorisuskop/96/4  .......... .......... .......... .......... .......... .......... .......... .......... 716
Zaire               .......... .......... .......... .......... .......... .......G.. .......... .......... 716
Warthog             .......... .......... .......... .......... .......... .......... .......... .......... 716
Tengani_62          .......... .......... .......... .......... .......... .......... .......... .......... 716
MAL/19/Karonga      .......... .......... .......... .......... .......... .......... .......... .......... 720
GZ201801            .......... .......... .......... .......... .......... .......... .......... .......... 720
ASFV-G              .......... .......... .......... .......... .......... .......... .......... .......... 720
ASFV_Wuhan_2019-1   .......... .......... .......... .......... .......... .......... .......... .......... 716
E75                 .......... .......... .......... .......... .......... .......... .......... .......... 720
OURT_88/3           .......... .......... .......... .......... .......... .......... .......... .......... 720
Benin_97/1          .......... .......... .......... .......... .......... .......... .......... .......... 716
K49                 .......... .......... .......... .......... .......... .......... .......... .......... 720
Mkuzi_1979          .......... .......... .......... .......... .......... .......... .......... .......... 716
OmLF2               .......... .......... .......... .......... .......... .......... .......... .......... 716
Ken.rie1            .......... .......... .......... .......... .......... .......... .......... .......... 716
Uvira_B53           .......... .......... .......... .......... .......... .......... I......... .......... 716
BUR/18/Rutana       .......... .......... .......... .......... .......... .......... I......... .......... 716
Ken05/Tk1           .......... .......... .......... .......... .......... .......... .......... .......... 716
Kenya_1950          .......... .......... .......... .......... .......... .......... .......... .......... 716
Ken06.Bus           .......... .......... .......... .......... .......... .......... .......... .......... 716
Malawi_Lil-20/1     .......... .......... .......... .......... .......... .......... ....E..... .......... 716
Conservation

                           740                 760                 780                 800
G1211R_DR-1980      CHEKVLLSMK AMSTLCAEVN EYLRQDNGTS YLRMAYEEVL FPVCFTGKKK YYGIAHVNTP NFNTKELFIR GIDIIKQGQT 800
RSA_2_2008          .......... .......... .......... .......... .......... .......... .......... .......... 800
SPEC_57             .......... .......... .......... .......... .......... .......... .......... .......... 796
Warmbaths           .......... .......... .......... .......... .......... .......... .......... .......... 796
RSA_2_2004          .......... .......... .......... .......... .......... .......... .......... .......... 796
Pretorisuskop/96/4  .......... .......... .......... .......... .......... .......... .......... .......... 796
Zaire               .......... .......... .......... .......... .......... .......... .......... .......... 796
Warthog             .......... .......... ....R..... .......... .......... .......... .......... .......... 796
Tengani_62          .......... .......... .........N .......... .......... .......... .......... .......... 796
MAL/19/Karonga      .......... .......... .......... .......... .......... .......... .......... .......... 800
GZ201801            .......... .......... .......... .......... .......... .......... .......... .......... 800
ASFV-G              .......... .......... .......... .......... .......... .......... .......... .......... 800
ASFV_Wuhan_2019-1   .......... .......... .......... .......... .......... .......... .......... .......... 796
E75                 .......... .......... .......... .......... .......... .......... .......... .......... 800
OURT_88/3           .......... .......... .......... .......... .......... .......... .......... .......... 800
Benin_97/1          .......... .......... .......... .......... .......... .......... .......... .......... 796
K49                 .......... .......... .......... .......... .......... .......... .......... .......... 800
Mkuzi_1979          .......... .......... .......... .......... .......... .......... .......... .......... 796
OmLF2               .......... .......... .......... .......... .......... .......... .......... .......... 796
Ken.rie1            .......... .......... .......... .......... .......... .......... .......... .......... 796
Uvira_B53           .......... .......... .......... .......... .......... .......... .......... .......... 796
BUR/18/Rutana       .......... .......... .......... .......... .......... .......... .......... .......... 796
Ken05/Tk1           .......... ...I...... .......... .......... .......... .......... .......... .......... 796
Kenya_1950          .......... .......... .......... .......... .......... .......... .......... .......... 796
Ken06.Bus           .......... .......... .......... .......... .......... .......... .......... .......... 796
Malawi_Lil-20/1     .......... .......... .......... .......... .......... .......... .......... .......... 796
Conservation
```

G1211R Alignment

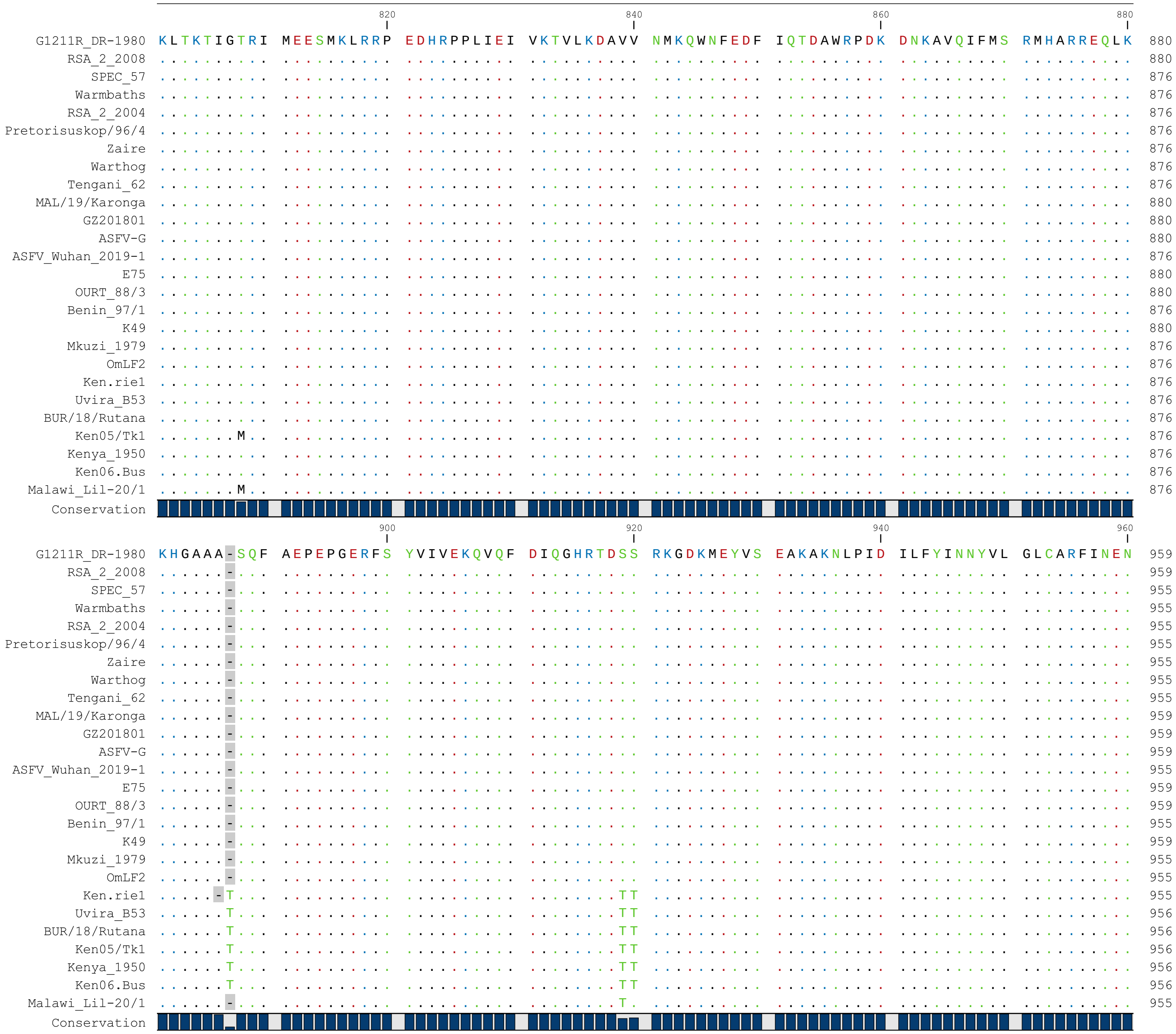

G1211R Alignment

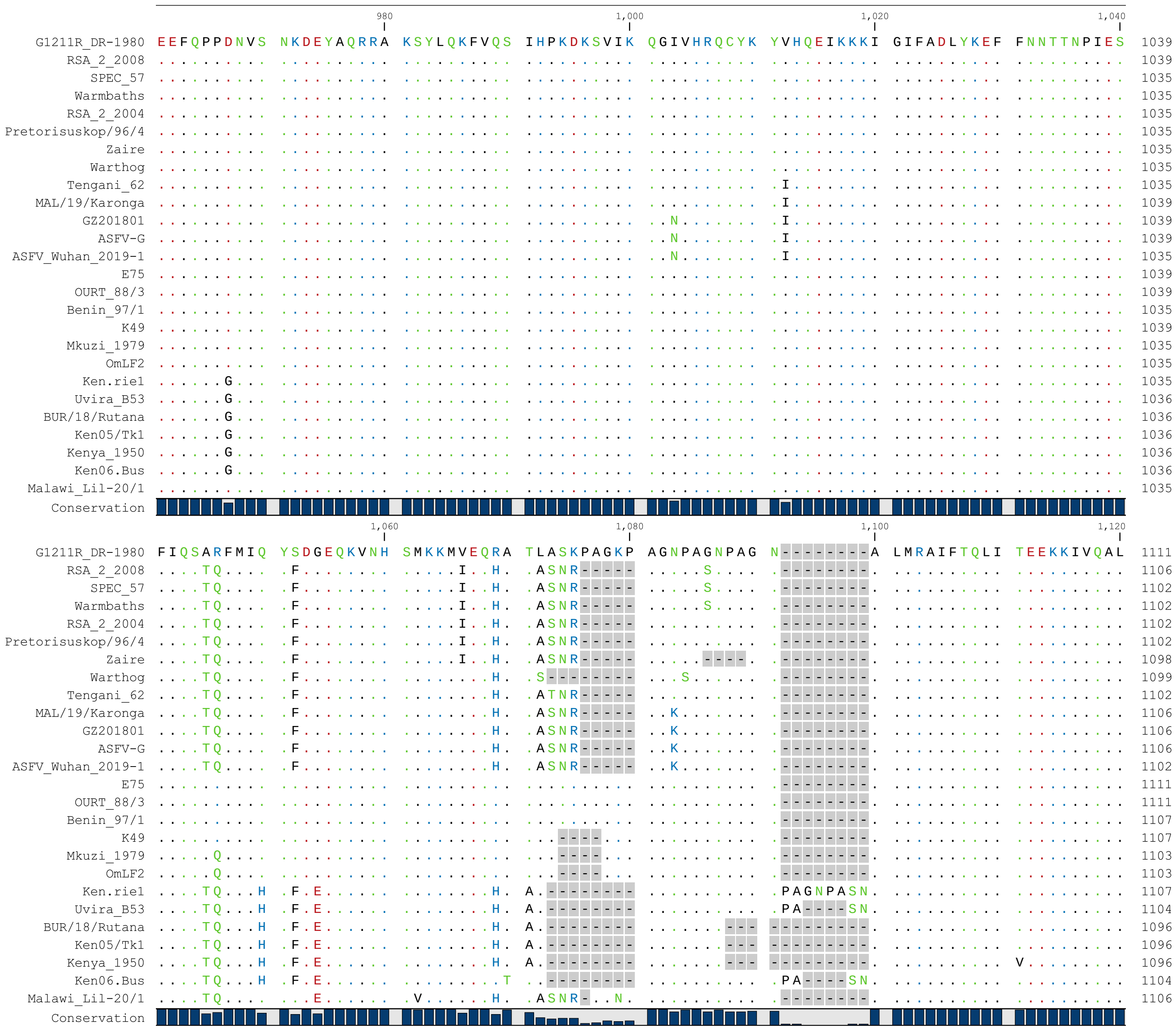

G1211R Alignment

```
                                         1,140                1,160                1,180                1,200
G1211R_DR-1980        YNKGDAIHDL LTYIINNINY KIATFQTKQM LTFEFSSTHV ELLLKLNKTW LILAGIHVAK KHLQALLDSY NNEPPSRTFI 1191
RSA_2_2008            .......... .......... .......... .......... .......... .......... .......... .......... 1186
SPEC_57               .......... .......... .......... .......... .......... .......... .......... .......... 1182
Warmbaths             .......... .......... .......... .......... .......... .......... .......... .......... 1182
RSA_2_2004            .......... .......... .......... .......... .......... .......... .......... ......K... 1182
Pretorisuskop/96/4    .......... .......... .......... .......... .......... .......... .......... ......K... 1182
Zaire                 .......... .......... .......... .......... .......... .......... .......... ......K... 1178
Warthog               .......... .......... .......... .......... .......... .......... .......... .......... 1179
Tengani_62            .......... .......... .......... .......... .......... .......... .....F.... ......K... 1182
MAL/19/Karonga        .......... .......... .......... .......... .......... .......... .....F.... ...S...... 1186
GZ201801              .......... .......... .......... .......... .......... .......... .....F.... ...S...... 1186
ASFV-G                .......... .......... .......... .......... .......... .......... .....F.... ...S...... 1186
ASFV_Wuhan_2019-1     .......... .......... .......... .......... .......... .......... .....F.... ...S...... 1182
E75                   .......... .......... .......... .......... .......... .......... .......... .......... 1191
OURT_88/3             .......... .......... .......... .......... .......... .......... .......... .......... 1191
Benin_97/1            .......... .......... .......... .......... .......... .......... .......... .......... 1187
K49                   .......... .......... .......... .......... .......... .......... .......... .......... 1187
Mkuzi_1979            .......... .......... .......... .......... .......... .......... .......... .......... 1183
OmLF2                 .......... .......... .......... .......... .......... .......... .......... .......... 1183
Ken.rie1              .....E.... .S........ .......... ....L....I .......... ...V...... ...HV..GLS ......K... 1187
Uvira_B53             .....E.... .......... .......... ....L..... .......... ...V...... ...HV..GLS ......K... 1184
BUR/18/Rutana         .....E.... .......... .......... ....L..... .......... ...V...... ...HV..GLS ......K... 1176
Ken05/Tk1             .....E.... .......... .......... ....L..... .......... ...V...... ...HV..GLS ......K... 1176
Kenya_1950            .....E.... .......... .......... ....L..... .......... ...V...... ...HV..GLS ......K... 1176
Ken06.Bus             .......... .S........ .......... ....L..... .......... ...V...... ...HT..GLS ......K... 1184
Malawi_Lil-20/1       .S...E.... .......... .......... ....L..... .......... ...T...... .......... ......K... 1186
Conservation

                                         1,220
G1211R_DR-1980        QQAIEEECGS IKPSCYDFIS 1211
RSA_2_2008            .......... .......... 1206
SPEC_57               .......... .......... 1202
Warmbaths             .......... .......... 1202
RSA_2_2004            .......... .......... 1202
Pretorisuskop/96/4    .......... .......... 1202
Zaire                 .......... .......... 1198
Warthog               .......... .......... 1199
Tengani_62            .......... .......... 1202
MAL/19/Karonga        .......... .......... 1206
GZ201801              .......... .......... 1206
ASFV-G                .......... .......... 1206
ASFV_Wuhan_2019-1     .......... .......... 1202
E75                   .......... .......... 1211
OURT_88/3             .......... .......... 1211
Benin_97/1            .......... .......... 1207
K49                   .......... .......... 1207
Mkuzi_1979            .......... .......... 1203
OmLF2                 .......... .......... 1203
Ken.rie1              .......... .......... 1207
Uvira_B53             .......... .......... 1204
BUR/18/Rutana         .......... .......... 1196
Ken05/Tk1             .......... .......... 1196
Kenya_1950            .......... .......... 1196
Ken06.Bus             .......... .......... 1204
Malawi_Lil-20/1       ....K...S. .......... 1206
Conservation
```

I215L Alignment

```
                                         20                             40                             60                             80
I215L_DR-1980        MVSRFLIAEY RHLIENPSEN FKISVNENNI TEWDVILRGP PDTLYEGGLF KAKVAFPPEY PYAPPKLTFT SEMWHPNIYP  80
E75                  .......... .......... .......... .......... .......... .......... .......... ..........  80
OURT_88/3            .......... .......... .......... .......... .......... .......... .......... ..........  80
LO2018_minor         .......... .......... .......... .......... .......... .......... .......... ..........  80
K49                  .......... .......... .......... .......... .......... .......... .......... ..........  80
Arm/07/CBM/c4        .......... .......... .......... .......... ...F...... ...I...... .......... ..........  80
Warthog              ......M... .......... ........DM .......... .......... .......... .....R.... ..........  80
Mkuzi_1979           ......M... .......... .........M .......... .......... .......... .....R.... .........S  80
OmLF2                ......M... .......... .......... .......... .......... .......... .....R.... .........S  80
Warmbaths            ......L... .......... ........DM .......... .......... .......... .....R.... ..........  80
Georgia_2007/1       .......... .......... .......KDM .......... ...F...... ...I...... .....R.... .........S  80
Tengani_62           ......M... .......... ..V......L .......... ...F...... .......... .....R.... .........S  80
Pretorisuskop/96/4   ......M... ...T...... ..V......M .......K.. A......... ...IG...N. ..E..R.... .........S  80
Malawi_Lil-20/1      ...S..L... KN..V....H .......D.L .......K.. .......... ...IV...K. ..E..R.... .........S  80
Ken05/Tk1            ......LT.. .N.T.....H ..V......L .......K.. ...F...... ...IT...N. ..E....... ..........  80
Ken05/Tk1            ......LT.. .N.T.....H ..V......L .......K.. ...F...... ...IT...N. ..E....... ..........  80
Ken.rie1             ......LT.. .N.T.....H ..V......L .......K.. ...F...... ...IT...N. ..E....... ..........  80
Uvira_B53            ......LT.. .N.T.....H ..V......L .......K.. ...F...... ...IT...N. ..E....... ..........  80
BUR/18/Rutana        ......LT.. .N.T.....H ..V......L .......K.. ...F...... ...IT...N. ..E....... ..........  80
Kenya_1950           ......LT.. .N.T.....H ..V......L .......K.. S..F...... ...IT...N. ..E....... ..........  80
Ken06.Bus            ......L... .N.T...... ..V......L .......K.. ...F...... ...IN...N. ..E......I .........A  80
Conservation

                                        100                            120                            140                            160
I215L_DR-1980        DGRLCISILH GDNAEEQGMT WSPAQKIDTI LLSVISLLNE PNPDSPANVD AAKSYRKYVY KEDLESYPME VKKTVKKSLD 160
E75                  .......... .......... .......... .......... .......... .......... .......... .......... 160
OURT_88/3            .......... ....G..... .......... .......... .......... .......... .......... .......... 160
LO2018_minor         .......... .......... .......... .......... .......... .......C.. .......... .......... 160
K49                  .......... .......... .......... .......... .......... ......R... .......... .......... 160
Arm/07/CBM/c4        .......... .......... .......... .......... .......... .......LL. .......... .......... 160
Warthog              ..K....... .......... .......... .......... .......... .......LMH .......... ..R....... 160
Mkuzi_1979           ..K....... .......... .......... .......... .......... .......LMH .......... ..R....... 160
OmLF2                ..K....... .......... .......... .......... .......... ......R..H .......... .......... 160
Warmbaths            ..K....... .......... .......... .......... .......... .......FL. .......... .......... 160
Georgia_2007/1       ..K....... .......... .......... .......... .......... .......LL. .......... ..R....... 160
Tengani_62           ..K....... .......... ...V.....V .......... .......... ........L. .......... ..R....... 160
Pretorisuskop/96/4   ...V...... .......... ...V...... .......... ..T....... ......RF.H .......... ..R....... 160
Malawi_Lil-20/1      ..K....... .......... .........V .......... .......... ........L. .......... .......... 160
Ken05/Tk1            .......... T.....R... .......E.V .......F.. ..T....... .......LMH .......... .......... 160
Ken05/Tk1            .......... T.....R... .......E.V .......F.. ..T....... .......LMH .......... .......... 160
Ken.rie1             .......... T..V..R... .......E.V .......F.. ..T....... .......LMH .......... .......... 160
Uvira_B53            .......... T..V..R... .......E.V .......F.. ..T....... .......LMH .......... .......... 160
BUR/18/Rutana        .......... T..V..R... .......E.V .......F.. ..T....... .......LMH .......... .......... 160
Kenya_1950           .......... T..V..R... .......E.V .......F.. ..T....... .......LMH .......... .......... 160
Ken06.Bus            .......... T..P..R... .......E.V .......F.. ..T....... .......FMH .......A.. .......... 160
Conservation

                                        180                            200                            220
I215L_DR-1980        ECSPEDIEYF KNAASNVPPI PSDAYED--E CEEMEDDTYI LTYDD----- --EEEEDEEM DDE 214
E75                  .......... .......... .......--. .......... .....----- -D........ ... 215
OURT_88/3            .......... .......... .......--. .......... .....----- -D........ ... 215
LO2018_minor         .......... .......... .......--. .......... .....----- -D........ ... 215
K49                  .......... .......... .......--. .......... .....----- -D...-.... ... 214
Arm/07/CBM/c4        .......... .......... .......--. .......... .....----- -E........ ... 215
Warthog              .......... ...V....A. .......--. .......... .....EDEEE ED........ ... 221
Mkuzi_1979           .......... .......... .......--. .......... .....----- -D........ ... 215
OmLF2                .......... .......... .......--. .......... .....----- -D........ ... 215
Warmbaths            .......... ..V....... .......--. ......G... .....E---- -D........ ... 216
Georgia_2007/1       .......... .......... .......--. .......... .....----- ----.DE..E ... 212
Tengani_62           .......... .....D.... .......--. .......... .....----- --........ ... 214
Pretorisuskop/96/4   .......... .......... .......--. S......... .....ED--- -E........ ... 217
Malawi_Lil-20/1      ...A...... ..VPV..L.V ...D...--. --....G... .....----- -ED....... ... 213
Ken05/Tk1            ...A...... ...VF...A. ......--H. D......... .....----- ED........ ... 216
Ken05/Tk1            ...A...... ...VF...A. ......--H. D......... .....----- ED........ ... 216
Ken.rie1             ...A...... ...VF...A. ......--H. D......... .....----- ED........ ... 216
Uvira_B53            ...A...... ...VF...A. .......EH. D.....G... .....----- ED.....D.. ... 218
BUR/18/Rutana        ...A...... ...VF...A. ......--H. D.....G... .....----- ED.....D.. ... 216
Kenya_1950           ...A...... ...VF...A. .......EH. D.....G... .....----- ED.....D.. ... 218
Ken06.Bus            ...A...... ...TF...A. ......--H. D......... .....D---- ED........ ... 217
Conservation
```

Alignment of DP93R & KP93L

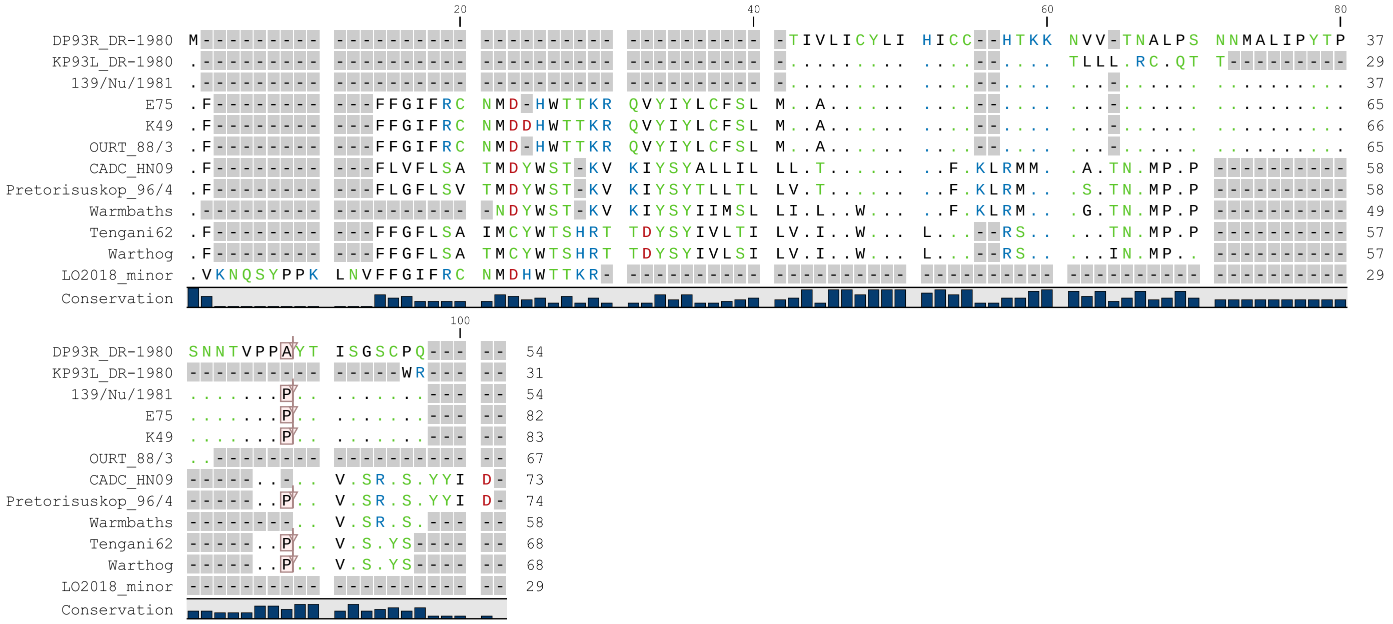

L60L Alignment

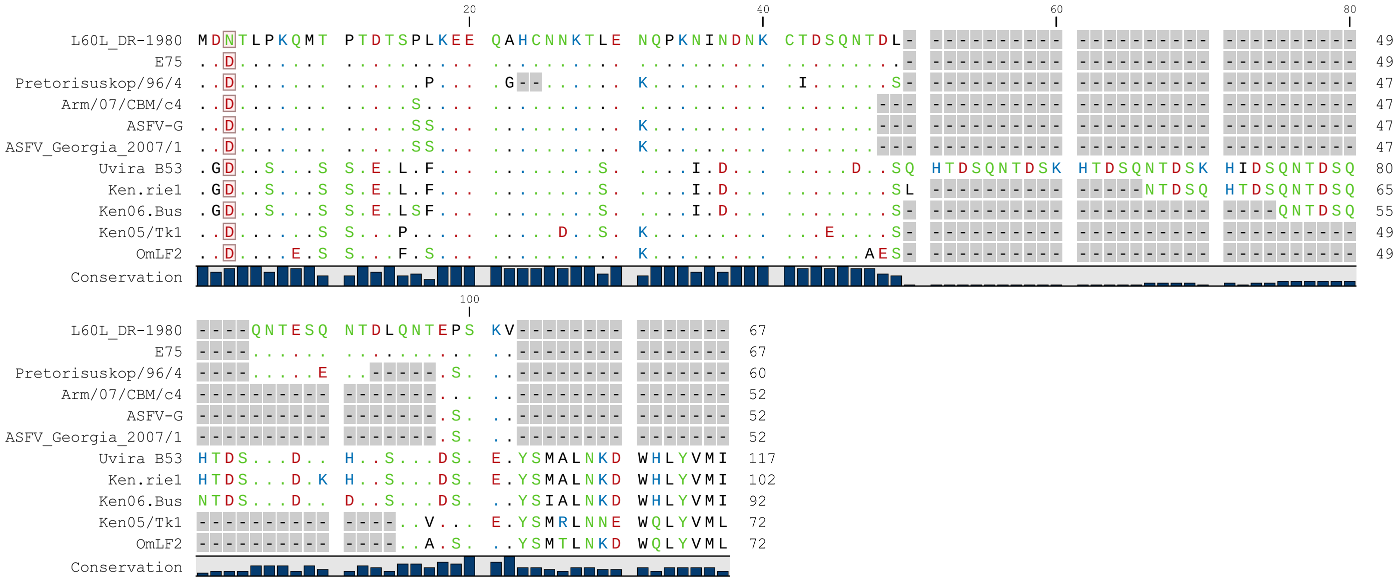

MGF360-12L Alignment

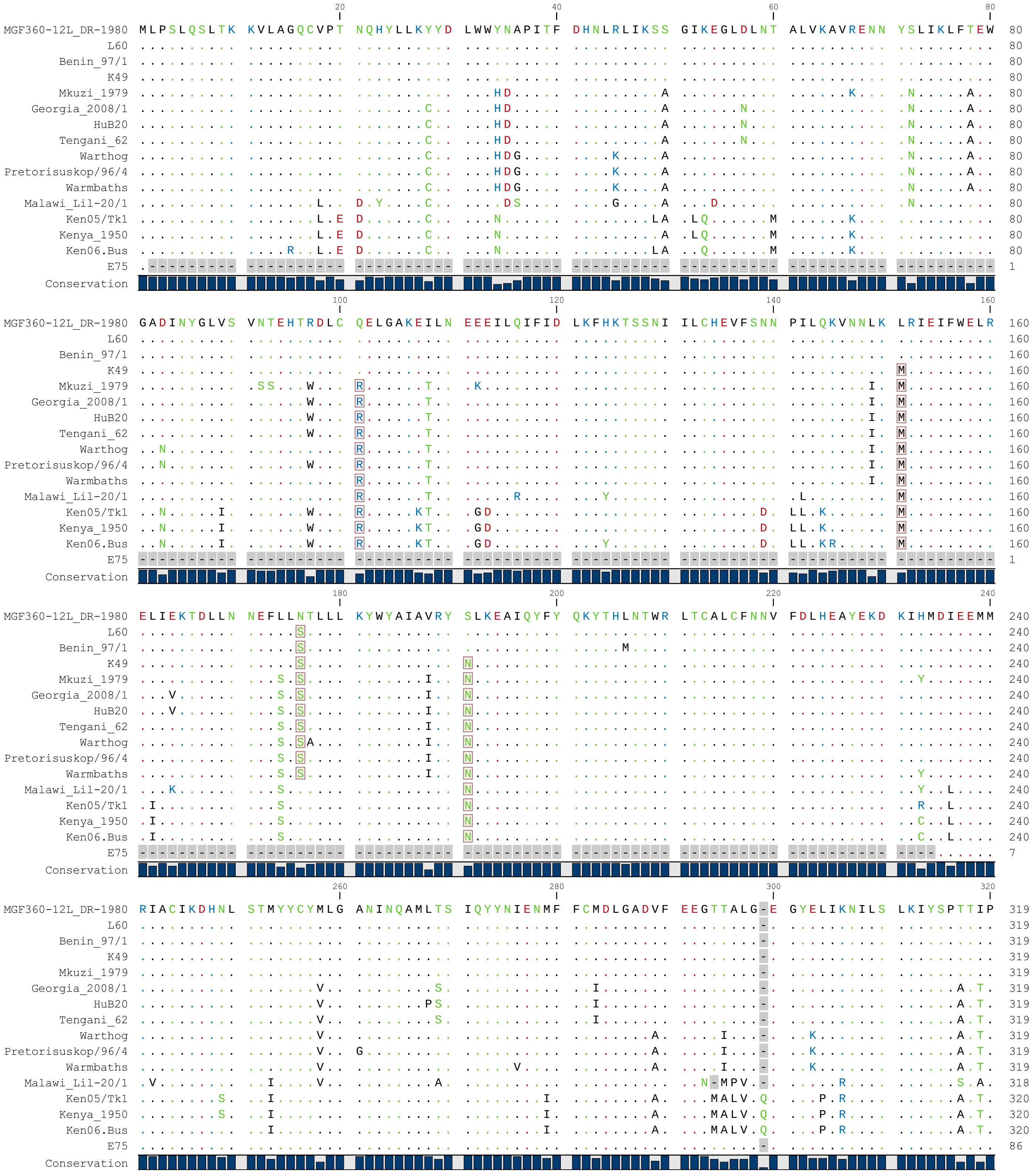

MGF360-12L Alignment

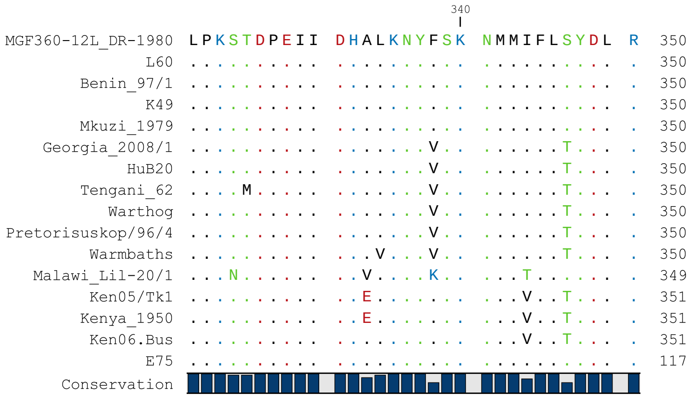

Supplemental Figure 1: Amino acid sequence alignments of CP2475L, G1211R, I215L, KP93L & DP93R, L60L, and MGF 360-12L originating from various sequenced isolates and DR-1980. Unique amino acids within the DR-1980 isolate are highlighted.
