# Supplementary material for: Full genome sequence for the African swine fever virus outbreak in the Dominican Republic in 1980
Source: Sci Rep. 2023 Jan 19;13:1024. doi: 10.1038/s41598-022-25987-5 (PMC9852453; doi:10.1038/s41598-022-25987-5)
Supplement: Supplementary file 2 — Supplementary Figure 2. [file 41598_2022_25987_MOESM2_ESM.pdf]

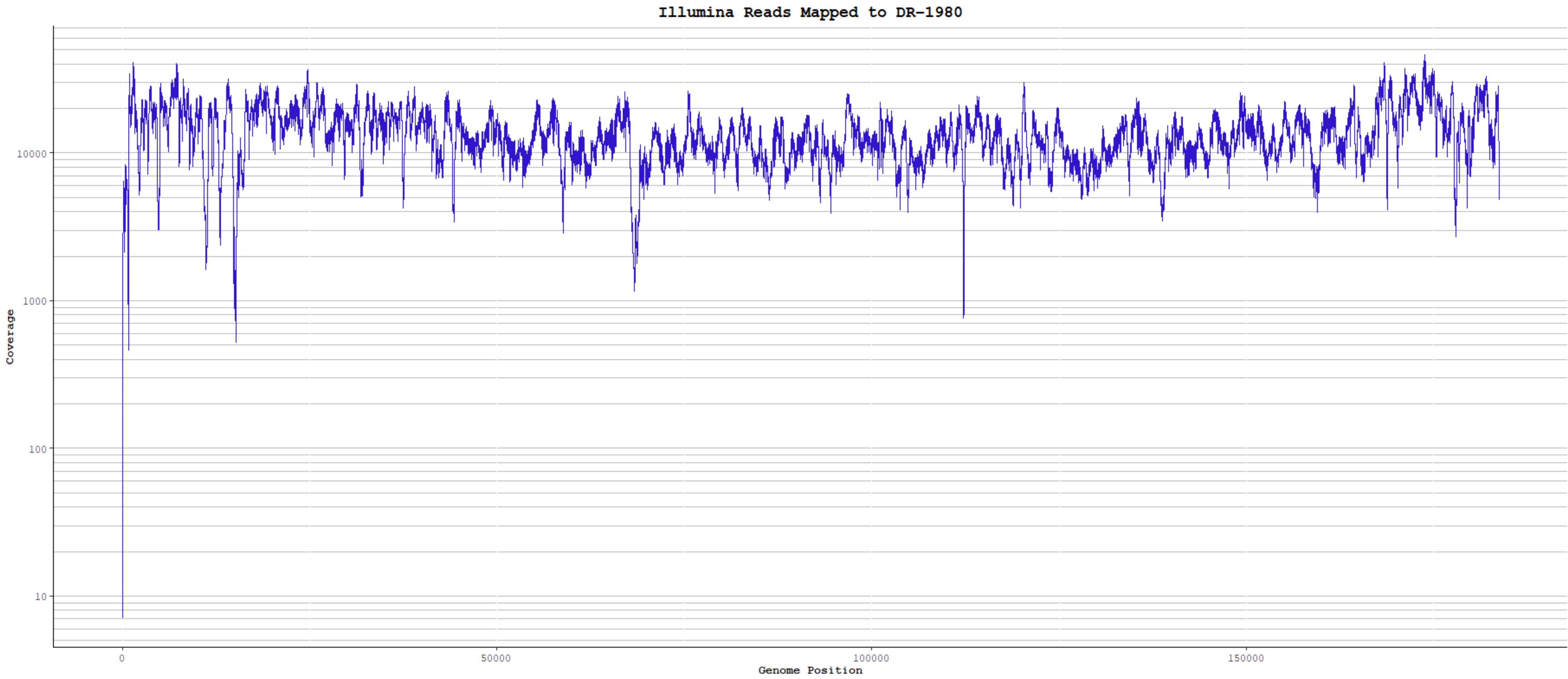

Supplemental Figure 2. Trimmed Illumina reads were mapped backed to the DR-1980 genome using the default parameters of the Map Reads to Contigs module of CLC Genomics Workbench. The depth of coverage (y) is indicated at each position of the genome (x).
